# An Improved Vector System for Homogeneous and Stable Gene Regulation

**DOI:** 10.3390/ijms22105206

**Published:** 2021-05-14

**Authors:** Barbara Michalec-Wawiórka, Jakub Czapiński, Kamil Filipek, Patrycja Rulak, Arkadiusz Czerwonka, Marek Tchórzewski, Adolfo Rivero-Müller

**Affiliations:** 1 Department of Molecular Biology, Institute of Biological Sciences, Maria Curie-Skłodowska University, 20-033 Lublin, Poland; kamilfilipek.zbm@gmail.com (K.F.); patrycjarulak@wp.pl (P.R.); maro@hektor.umcs.lublin.pl (M.T.); 2 Department of Biochemistry and Molecular Biology, Medical University of Lublin, 20-093 Lublin, Poland; jakubczapinski@umlub.pl (J.C.); arkadiuszczerwonka@umlub.pl (A.C.); adolfo.rivero-muller@umlub.pl (A.R.-M.); 3 Postgraduate School of Molecular Medicine, 02-091 Warsaw, Poland

**Keywords:** gene regulation, expression systems, Tet-On, ribosomal proteins, uL10 (P0)

## Abstract

Precise analysis of the genetic expression and functioning of proteins requires experimental approaches that, among others, enable tight control of gene expression at the transcriptional level. Doxycycline-induced Tet-On/Tet-Off expression systems provide such an opportunity, and are frequently used to regulate the activity of genes in eukaryotic cells. Since its development, the Tet-system has evolved tight gene control in mammalian cells; however, some challenges are still unaddressed. In the current set up, the establishment of the standard Tet-based system in target cells is time-consuming and laborious and has been shown to be inefficient, especially in a long-term perspective. In this work, we present an optimized inducible expression system, which enables rapid generation of doxycycline-responsive cells according to a one- or two-step protocol. The reported modifications of the Tet-On system expand the toolbox for regulated mammalian gene expression and provide high, stable, and homogenous expression of the Tet-On3G transactivator, which is of fundamental importance in the regulation of transgenes.

## 1. Introduction

The development of experimental biological sciences is only possible owing to the engineering of new molecular tools and their continuous improvement, which provide more reliable insight into the functioning of living cells. For instance, the regulation of the expression of a gene of interest (GOI) in a quantitative and temporal manner contributes to the understanding of gene functioning and interdependence between its protein product and cellular metabolism. Tight gene expression regulation is of great importance especially if the GOI induces cytotoxicity, leads to cell cycle arrest or apoptosis, or induces differentiation. There are many inducible expression systems designed for mammalian cells like the tetracycline-controlled operator system [1], the cumate-controlled operator system [2], protein–protein interaction-based chimeric systems [3], tamoxifen-controlled recombinase systems [4], and riboswitch-regulatable expression systems [5] (reviewed in [6]). Among these, the tetracycline-inducible systems are probably the most widespread among laboratories due to their efficiency and stringent regulation [7].

In one of its variants, i.e., the Tet-On inducible system, the transcription of a GOI is reversibly turned on in the presence of a synthetic tetracycline derivative—doxycycline (Dox). This system is based on two components: the Tet transactivator (TET3G), a protein that structurally responds to Dox, and the Tet-binding promoter upstream of the GOI. Dox binds the transactivator and induces its conformational changes that promote binding to the tet operator (tetO) sequence placed upstream a minimal promoter, which results in downstream transcription of the GOI. The critical step here is to obtain a double stable cell line with a constant and stable level of transactivator expression, which is persistent over time and efficiently transactivates the GOI upon doxycycline treatment in a dose-dependent manner. Yet, the generation of double stable cell lines based on currently available systems is time-consuming and inefficient and requires at least two rounds of selection and screening. The first step is dedicated for screening of clones with stable expression of the transactivator, non-detectable basal expression, and a high homogenous level of induction upon Dox dosing. The second step is another round of clone selection for those also having the GOI. Many expression systems use separate promoters for the transactivator and the selection marker, which frequently leads to false positive clones and heterogeneous populations. In addition, the commonly used neomycine resistance (*Neo^R^*) is characterized by a long selection period, which can last from 2 to 4 weeks and, despite this time, still might be inefficient. All this results in poor efficiency, as not every resistant clone has transactivator expression, and those expressing it will have different levels of expression.

The next drawback of some of the common inducible expression systems is that the transactivator is under the control of the human cytomegalovirus (CMV) immediate-early promoter/enhancer. Although the CMV promoter is one of the most frequently used in vitro and in vivo promoters for driving the expression of transgenes in mammalian cells, due to its strong promoter activity, it has been found to be epigenetically silenced over time [8,9]. This feature is especially undesirable in carefully sorted stable cell lines, where permanent and constant expression of the transactivator guarantees efficient induction of the GOI. The invariability in time of selected clones with defined transactivator expression characteristics is also important for practical reasons. Stable clones with the most desirable qualities, once developed, can be cryopreserved and banked for further use e.g., to obtain subsequent cell lines with regulated expression of novel GOIs, without the need to restart the entire procedure from the very beginning every time.

Here, we describe a modified inducible gene expression system, which provides several advantages over the standard Tet-On system. The improved tool significantly increases the efficiency of generation of stable cell lines and shortens the time of the whole procedure, which provides fast selection of desired clones, persistent over time, high and invariant expression of the transactivator and thus leads to successful downstream regulation of GOI. As a proof of principle, we have generated a cell line with tightly regulated expression of ribosomal uL10 protein (formerly known as P0 [10]), which is involved in translation per se and also displays potential to regulate other processes [11,12]. Importantly, dysregulation of uL10 expression has been associated with tumorigenesis [13,14,15,16,17,18]; therefore obtaining a tightly tunable expression system made possible to build a robust cellular model to trace the relationship between uL10 and cell abnormalities and proved the applicability of our new protocol to physiologically relevant targets.

## 2. Results

### 2.1. Designing A New Variant of the Mammalian Inducible Expression System

**Figure 1:**
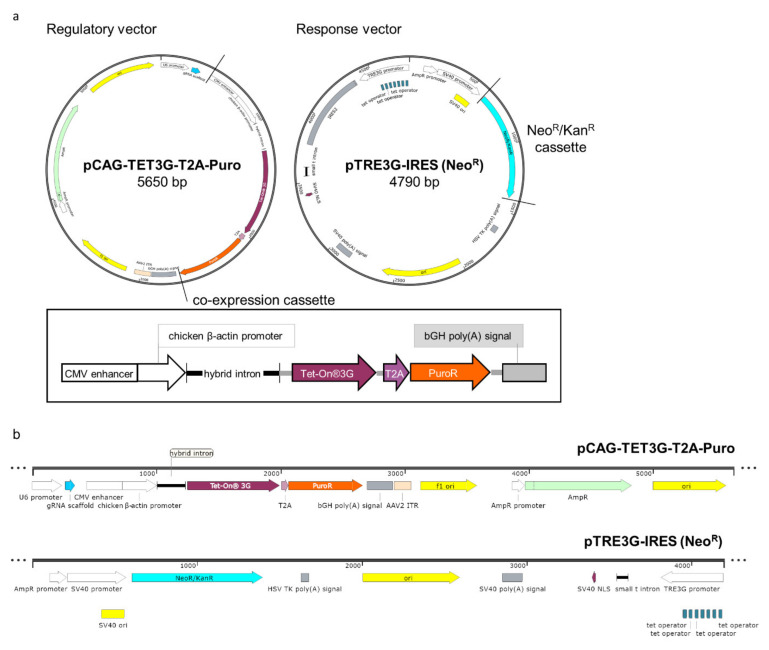
Overview of the redesigned inducible expression system. Genetic maps of regulatory and response vectors with introduced modifications, presented in circular (**a**) and linear (**b**) views, were prepared with SnapGene (SnapGene^®^ software from GSL Biotech; available at snapgene.com). The re-engineered transactivator system involved the cloning of the chicken β-actin (CAG) promoter (CAGp) upstream the transactivator gene (*TET3G*), followed by T2A and the puromycin (Puro) resistance gene (*PuroR*) to ensure equal co-expression of TET3G and Puro, and is marked on the regulatory vector backbone with black lines and enlarged in the box as a co-expression cassette (**a**). The response vector was modified by cloning a cassette containing the *Kan/Neo* resistance gene, indicated on the vector backbone with cyan color, under a double promoter (eukaryotic/prokaryotic) (**a**,**b**).

Having found that the standard inducible expression system was not sufficiently satisfactory for the generation of cell lines that express the transactivator stably, efficiently, and homogenously and that the development of double stable cell lines according to the original protocol requires considerable amounts of time and labor, we have re-engineered the system and modified both regulatory (pCMV-TET3G) and response (pTRE3G-IRES) plasmids (Figure 1) from the Tet-On^®^3G system (Takara).

The regulatory plasmid was recreated to solve two of the main problems of the standard system: the poor efficiency in establishing inducible cell lines and the instability of generated lines over time. To address these limitations, firstly we came across the idea to fuse the transactivator gene with the puromycin resistance gene (*PuroR*), separated by a bi-cistronic T2A self-cleaving peptide sequence. This ensures that puromycin (Puro)-resistant cells also express the transactivator in a 1:1 ratio and provides fast and effective antibiotic selection of transactivator-positive clones. Secondly, we exchanged the cytomegalovirus promoter (CMVp) for that of chicken β-actin upon a minimal CMV promoter (CAGp). The CMVp is known to be frequently epigenetically silenced [8,9], while CAGp contributes to persistent and high-level expression of a transgene [19,20]. The regulatory plasmid was constructed with the three-fragment Gibson assembly used to introduce the chicken β-actin promoter and the transactivator (TET3G) into the 2A-Puro vector, PX459. The three fragments were amplified by PCR using primers containing >15 nts homology arms (primers in Supplementary Section Table S1). The resulting vector, named pCAG-TET3G-T2A-Puro regulatory vector, is shown in Figure 1. The full sequence is presented in the Supplementary Section as in Addgene (#158067).

In the standard method, the preparation of a cell line having both the transactivator and the Dox-regulated GOI vectors relies on the second round of drug selection after transfection of the cell line stably expressing the transactivator with the Dox-responsive conditional vector (containing the GOI) and a linear selection marker. To make this procedure more straightforward, we also modified a response plasmid, which later on enabled us to propose a one-step protocol for a generation of inducible cell lines. For that, we inserted the Neo^r^ cassette into the response plasmid backbone (Figure 1) using REPLACR mutagenesis [21] and prepared two vectors to validate our system functionally. In one of them, the GOI was a reporter gene (*EGFP*)— coding GFP (pTRE3G-EGFP-IRES, from here onwards abbreviated as pTRE3G-GFP, Supplementary Figure S1), while the second was carrying a biologically significant gene (*RPLP0*) — coding the human ribosomal protein uL10 fused with GFP at its N-terminus (pTRE3G-EGFP-uL10-IRES, abbreviated as pTRE3G-GFP-uL10, Supplementary Figure S1). The sequences of both vectors are placed in the Supplementary Section and can be found in Addgene (#158068 and #158069). These two vectors, like their predecessors, are bi-cistronic with IRES sequences allowing efficient co-expression of the two GOIs.

### 2.2. Fast Antibiotic Selection and Efficient Clone Isolation 

To test the efficiency of the re-designed system, we followed the general protocol provided by the manufacturer of the standard system and established two stable HEK293 (Human Embryo Kidney) cell lines expressing the transactivator, either using the original (Neo^R^) or our puromycin regulatory vector. For that, HEK293 cells were seeded on single wells of 24-well plates at the density of 5 × 10^4^ cells per well, cultured for 24 h, and transfected with either the standard pCMV-TET3G(Neo^R^) from the Tet-On^®^ 3G Inducible Expression System (Takara) or the modified regulatory plasmid pCAG-TET3G-T2A-Puro for constitutive transactivator expression. After 48 h, the cells were split into 3 × 10 cm Petri dishes and grown for another 48 h, when selective antibiotics: geneticin (G418) at a concentration of 1200 µg/mL or puromycin at a concentration of 0.5 µg/mL (low) or 1.2 µg/mL (high) were added, respectively. Antibiotic resistant cell colonies appearing after the G418/Puro-selection periods were isolated with cloning cylinders and transferred into separate wells in 24-well plates. In each case, more than 20 clones were picked for further cultivation and testing. The first important observation was that single puromycin-resistant cells appeared just after four days, whereas the selection of G418-resistant clones required over two weeks.

**Figure 2:**
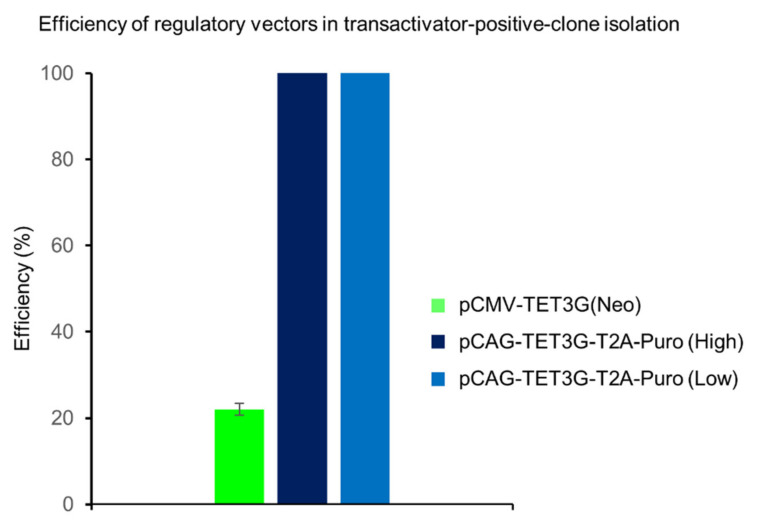
Efficiency of the newly designed system for inducible gene expression. The TetOn-3G-T2A-Puro-based system provides four times higher efficiency in isolating clones that express the transactivator stably vs. the standard Tet-On 3G system (Takara). HEK293 (Human Embryo Kidney) cells were transfected with either pCMV-TET3G(Neo^R^) or reengineered pCAG-TET3G-T2A-Puro, followed by treatment with selection antibiotics, geneticin (G418) and Puro, respectively. Efficiency was monitored for one concentration of G418 (1200 µg/mL) and two concentrations of Puro: low (0.5 µg/mL) and high (1.2 µg/mL). For each system, more than 20 single cell clones resistant to the selection agents were isolated in two independent biological replicates and 11 from those that survived were cultured and transfected with a response plasmid coding GFP under a Dox inducible promoter. The Dox-induced expression of GFP was monitored under a fluorescence microscope. Efficiency is presented in “%” and describes how many clones, among all that survived the isolation, expressed the GFP transgene in response to the Dox treatment. The data is presented as mean ± standard deviation (SD) from two independent experiments.

Single cell clones that survived the selection, isolation, and expansion were screened for their ability to induce GOI expression upon transfection with a GFP responsive plasmid (pTRE3G-GFP). After Dox (500 ng/mL) administration, the GFP-expressing clones were counted under a confocal fluorescence microscope and compared with the total number of isolated and analyzed resistant clones, which allowed calculation of efficiency presented in percentages (Figure 2). In this way, we showed that 100% of the clones with pCAG-TET3G-T2A-Puro responded to Dox, regardless of whether low (0.5 µg/mL) or high (1.2 µg/mL) puromycin concentrations were used for selection, whereas only 22% of G418-resistant clones with the original pCMV-TET3G(Neo^R^) exhibited Dox-induced response and GFP expression. These results underscore the advantage of having the transactivator in the same mRNA as a strong selection gene. We expected that the stronger selection (higher puromycin concentration) would generate cells expressing higher levels of the transactivator and thus higher levels of GFP upon Dox, but this was not the case; cells selected by the low or high concentrations of puromycin expressed identical levels of GFP, as determined by flow cytometry (Supplementary Figure S2). It is worth emphasizing that the puromycin-based selection and the application of the co-expression cassette significantly reduces the time and efforts involved in generation of cell lines with high and stable expression of the transactivator, a sine qua non for successful regulation of GOI. Isolated clones with the best features were regarded as cell lines that expressed the transactivator efficiently and were ready for creation of double stable cell lines with regulated GOI expression according to the standard two-step protocol based on subsequent antibiotic selection.

### 2.3. High Homogenous and Persistent Transactivator Expression

**Figure 3:**
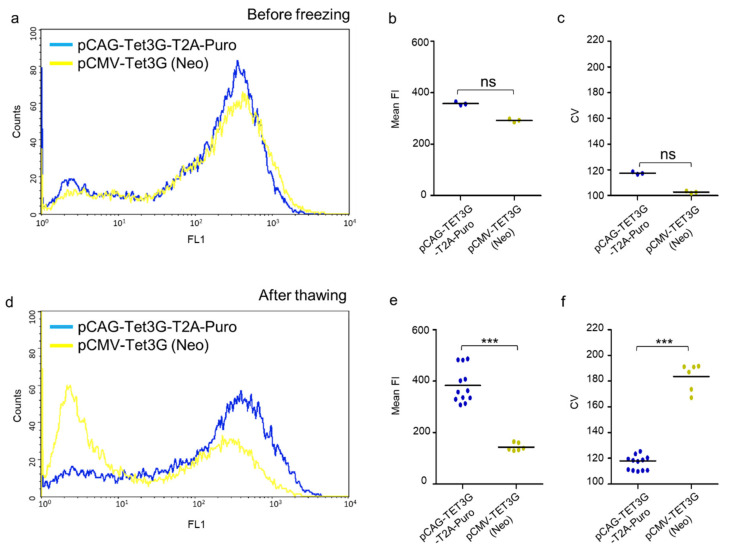
Characteristics of the transgene expression obtained with the new Tet-On3G-T2A-Puro system. The parameters of the transgene expression corresponding to the transactivator activity were measured by flow cytometry for both inducible HEK293 cell lines generated with redesigned TetOn-3G-T2A-Puro and original Tet-On3G systems before cryopreservation (**a**,**b**,**c**) and after long-term cryopreservation (>6 months), thawing, and cultivation (**d**,**e**,**f**). The stable cell line created with the new variant of the system shows twice higher mean fluorescence intensity of GFP transgene and a homogenous profile of its expression after the freezing–thawing cycle in comparison to the cell line established with the standard system. This is displayed on flow cytometry histograms (**a,d**) and reflected in the mean fluorescence intensity (FI) (**b,e**) and the coefficient of variation (CV) measurements (**c,f**) presented in the plots. Statistical significance of differences between both stable cell lines was calculated using the Mann–Whitney test and is represented by asterisks: *** *p* < 0.001; ns—not significant.

The homogeneity and stability of transactivator expression is a must, when establishing cell lines with regulated GOI. These expression features mostly depend on promotor performance and thus in the next step we investigated whether the CAGp is, unlike CMVp, insusceptible to silencing effects. To this end, both pCAG-TET3G-T2A-Puro and pCMV-TET3G(Neo^R^) stable cell lines obtained from single clones with a similar Dox-responsive pattern (no. 2 and 7, respectively) were analyzed immediately after selection and then after long-term cryopreservation (>6 months) and further cultivation (one month in the presence of selection agents). To avoid cofounding factors, such as the effects of the response plasmid, shortly after establishment the cell lines were transiently transfected with the pTRE3G-GFP vector and analyzed by flow cytometry after Dox induction of GFP expression. This reflected the condition of these lines at the starting point. The same procedure was performed for both stable cell lines after thawing and a period of recuperation. The resultant histograms showed a unimodal distribution of GFP expression for both cell lines before freezing (Figure 3a). After thawing, the distribution pattern of the GFP expression in the pCMV-TET3G(Neo^R^)-selected cells changed to a bimodal one, while in the pCAG-TET3G-T2A-Puro-resistant cells it stayed virtually equal as before freezing with one dominant population (Figure 3d), which revealed the invariability of this cell line in a long time perspective. The mean fluorescence intensity (FI) of the two cell populations was comparable before freezing (Figure 3b); however, after six months of cryopreservation and one month of culturing, the mean fluorescence of the cells with integrated pCMV-TET3G(Neo^R^) was reduced more than 2-fold in comparison to the cells with stably integrated pCAG-TET3G-T2A-Puro (Figure 3e). The next analyzed parameter was the coefficient of variation (CV), which should be regarded here as a measure of dispersion of the fluorescence intensity within the analyzed cells, equal to the standard variation (data dispersion) in the normal distribution of a bell curve and was used to provide information about the level of heterogeneity of populations. The inspection of CV revealed that, before freezing, both cell lines were similarly homogeneous with no statistical difference observed (Figure 3c). In turn, after the prolonged storage and re-culturing, the stable CMVp-dependent transactivator line was more heterogeneous in terms of the transgene expression profile, with a large non-fluorescent population (Figure 3d, left-hand peak), than the stable cell line created with our modified system (Figure 3f).

To summarize, many CMVp-TET3G cells did not respond to Dox induction after cryopreservation and re-cultivation, and those that did had decreased GFP fluorescence intensity and were more heterogeneous, in comparison to their original state (before freezing) or stable cells with our pCAG-TET3G-T2A-Puro improved system. Importantly, Puro-selected cells treated in the same way expressed GFP in a stable and homogenous manner and at an invariantly high level, which correlates with efficient transactivator expression and unchanged CAGp activity.

These results showed that the rearrangement introduced into the system for inducible gene expression provides high quality performance of cell lines with transactivator expression. Moreover, the system ensures high homogeneity of the cell population, which in turn allows immediate further tests without the need for single-cell clone selection.

### 2.4. One-Step Selection of Transactivator and Regulatable Vectors

To this point, the generation of double stable cell lines according to the standard protocol requires a two-step procedure—stable transactivator screening, followed by response vector transfection (Takara). We positively verified this approach also with our system and using the GFP response vector (Supplementary Figures S3 and S7). However, since our system has two different selection gene markers, one for each modified plasmid and already proved high-performance of transactivator-positive clones, we explored whether a one-step ‘fast-track’, protocol could be possible. Thus, instead of using the stable-transactivator-line, we at once co-transfected both modified plasmids, i.e., the regulatory and response plasmids, into ‘bare’ HeLa cells, followed by monitoring of Dox-induced GFP expression by confocal microscopy. As a proof of principle, this time we inserted a gene of biological interest encoding the human ribosomal protein uL10, fused with GFP, under the Tet-promoter (pTRE3G-GFP-uL10 response plasmid).

**Figure 4:**
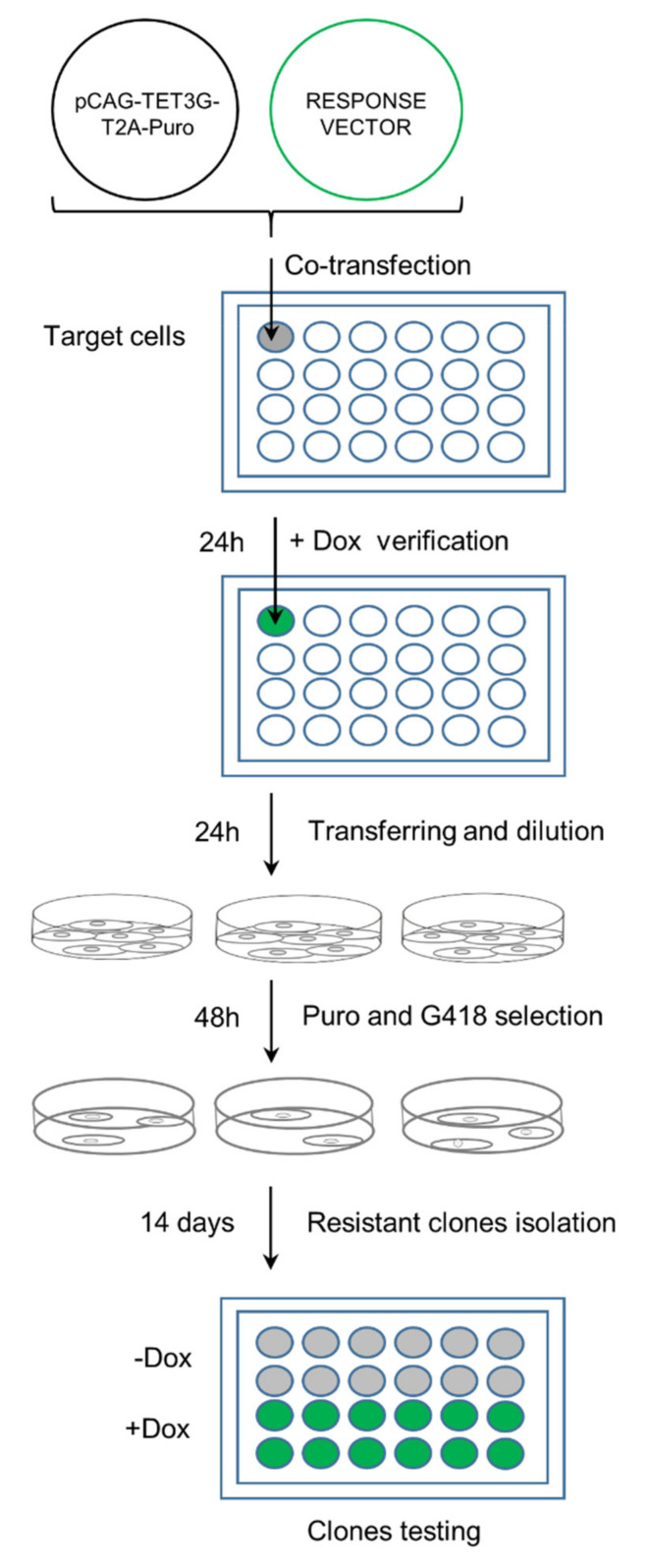
Scheme showing the workflow of the ‘fast-track’ protocol. Target cells were seeded on a 24-well plate and co-transfected with the regulatory and response plasmids in 1:1 ratio. After positive verification of the Dox-induced expression of GOI, cells from one well were transferred into three 10-cm dishes and, after the next 48 h, a combined selection with Puro and G418 was introduced. Resistant clones were isolated with cloning cylinders after 21 days, cultured, and tested for Dox inducibility in a 24-well plate format.

The detailed protocol, schematically shown in Figure 4, is as follows. The double stable cell lines generated according to the ‘fast track’ protocol were prepared by co-transfection of the modified regulatory, pCAG-TET3G-T2A-Puro, and response pTRE3G-GFP-uL10 vectors into HeLa cells seeded on a 24-well plate at the density of 5 × 10^4^ cells per well. For co-transfection, 1:1 ratios of the regulatory to response vectors were used (0.5 µg/mL: 0.5 µg/mL of DNA, respectively). 24 h post transfection, Dox (500 µg/mL) was applied to the cell culture. 24 h later, the cells were monitored for uL10-GFP expression under a fluorescence microscope. Upon the doxycycline treatment, uL10-GFP expression was observed within the cytoplasmic compartment (Supplementary Figure S4), which corresponds with its presence on ribosomal particles and is a standard distribution of the protein in steady-state conditions [11]. This result confirmed that both plasmids were working in a transient form. Next, the cells were split into 3 × 10 cm dishes and, after an additional 48 h, subjected to one-step antibiotic selection with Puro (0.5 µg/mL) and G418 (1000 µg/mL) combined together. The culture medium with the antibiotics was replaced every 72 h. Colonies of resistant clonal cells were isolated with cloning cylinders 21 days after the selection, transferred to fresh 24-well plates for further culturing, and monitored for uL10-GFP expression upon the Dox treatment. The uL10 protein has well-described subcellular localization and dynamics [11,12,22,23]; thus, it was easy to recognize positive clones expressing this transgene.

### 2.5. Characterization of GOI Expression in the Double Stable Cell Line Generated with the One-Step Protocol

**Figure 5:**
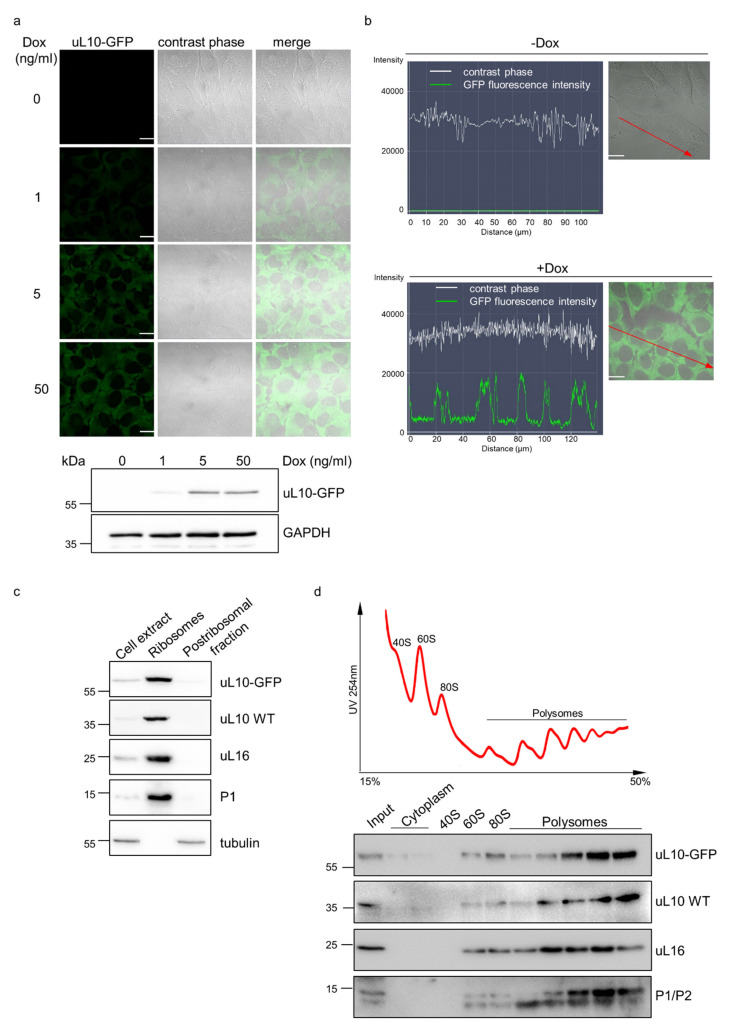
Characterization of Dox regulated expression of uL10-GFP in the double-stable HeLa cell line generated through the ‘fast track’ protocol. (**a**) Dox-dose dependent expression of uL10-GFP in the DS HeLa cell line established with the Tet-On3G-T2A-Puro system. *Left panel:* Live-cell images of double stable HeLa cells treated with different concentrations of Dox (0, 1, 5, 50 ng/mL). Fluorescence of uL10-GFP is shown on the left, the contrast phase view of the cells is shown in the middle panel, and merged images are presented on the right, scale bar 20 µm. *Right panel*: Immunodetection of uL10-GFP protein expression in response to different concentrations of Dox. Double stable HeLa cells were treated with increasing concentrations of Dox (0, 1, 5, 50 ng/mL) for dose-dependent induction of uL10-GFP, lysed and analyzed by Western blotting using anti-GFP antibodies. Antibodies against GAPDH were used as a loading control. Full-length blots are presented in Supplementary Figure S6a. (**b**) Fluorescence intensity profiles and live-cell images of DS HeLa cells after prolonged cryopreservation and cultivation upon Dox induction (+Dox) and without Dox (-Dox). Red arrows indicate the analyzed cells and correspond to the distance axis within the fluorescence intensity plots. Scale bar 20 µm. (**c**) Immunodetection of uL10-GFP and specified ribosomal proteins in post-nuclear cell extract, ribosomal (80S) and post-ribosomal (cytoplasmic) fractions of Dox-induced DS HeLa cells after prolonged storage and cultivation. Cells expressing uL10-GFP were fractionated and analyzed by Western blotting using anti-GFP antibodies and antibodies recognizing particular native ribosomal proteins: uL10, uL16, and P1. Antibodies against tubulin were used to show the purity of fractions. Full-length blots are presented in Supplementary Figure S6b. (**d**) Polysome profile of whole-cell polysomal extracts prepared from the Dox-induced DS HeLa cell line after prolonged storage and cultivation (*upper panel*) followed by Western blotting of polysomal fractions analyzed with antibodies against GFP and native ribosomal proteins: uL10, uL16, and P1 (*bottom panel*). Full-length blots are presented in Supplementary Figure S6c.

The resulting double stable (DS) cells were cultured as single clones. Next, after a month of continuous cultivation, one of such clones was tested for its dose response to Dox by confocal microscopy and Western blotting (Figure 5a). For that, doxycycline was removed from the medium to turn-off the expression of uL10-GFP. The cells were passaged three times and grown in Dox-free medium for a week to silence the expression of the analyzed protein. After that time, no background expression of GOI in the absence of Dox was observed, whereas administration of Dox at three different doses (1, 5, and 50 ng/mL) resulted in quantitative response of transgene expression, corresponding to the doxycycline concentration used until the system saturated with Dox above 5 ng/mL. Below this value, the higher the concentration of Dox that was applied, the higher the expression level of uL10-GFP that was observed, which reflects its tight and stable regulation.

To test the stability of the obtained DS cell line, uL10-GFP-expressing HeLa cells were cryo-preserved for 6 months and then thawed and cultivated for 20 passages. The confocal microscopy analysis of fluorescence intensity profiles showed that the DS HeLa cells preserved the ability to respond effectively and homogenously to Dox and expressed uL10-GFP after the Dox induction even after long-term storage and cultivation (Figure 5b). Next, using the same generation of cells, we analyzed the biological functionality of Dox-induced uL10-GFP in the DS HeLa cells and compared it with the behavior of the native uL10 ribosomal protein. Using cell fractionation and polysome profile analysis followed by immunodetection of uL10-GFP as well as native uL10 and other ribosomal proteins (RPs) used as controls, we showed that the GFP-fused uL10 protein expressed through our system was incorporated into ribosomal particles (Figure 5c) and was present in the population of actively translating ribosomes (polysomes) (Figure 5d). This resembled the behavior of native uL10 and other RPs and hence confirmed its involvement in translation. These results prove proper functioning of GFP-tagged uL10 in Dox-induced DS HeLa cells generated with the use of the one-step ‘fast track’ protocol.

Additionally, using the one-step protocol, we established our system in three other cell lines: HEK293, MEF (mouse embryonic fibroblasts), and NIH3T3 (mouse Swiss NIH embryonic fibroblasts) with similar results (Supplementary Figures S5 and S8), thus underpinning the universality of this approach.

Altogether, our results show that the modifications in the Tet-ON system enable fast and efficient generation of stable cell lines with tightly regulated GOI, but also with high homogenous and time-stable response. A one-step protocol was also established as an attractive alternative to the standard system procedure. The most important features of the redesigned system are summarized in Table 1.

## 3. Discussion

Since the discovery and initial design [1,24], the Tet-based system has undergone multiple modifications optimizing its operation in mammalian cells [25,26,27]. Nevertheless, despite the important improvements, e.g., modifications in the transactivator protein and tet-O promoter sequence that have brought tight regulation with no leaking expression of GOI in the absence of Dox and higher affinity and responsiveness in a Dox-dependent manner, the system still suffers from some limitations. In particular, it concerns a lengthy procedure to generate double cell lines and long-term instability of obtained clones. Here, we report a redesigned system to address these challenges. The main alteration proposed is the introduction of a T2A-linked bicistronic cassette providing stoichiometric and concordant expression of the transactivator and selectable marker (puromycin). T2A is a member of the 2A self-cleaving peptide class used in the expression of multiple proteins from a single open reading frame with high efficacy [28,29]. We showed that the implementation of the co-expression cassettes guaranteed that every antibiotic-resistant clone isolated during generation of a stable cell line expresses the Tet-On 3G transactivator. This approach overcomes the emergence of clones that are resistant to the selection agent but do not express the transactivator and, consequently, reduces the arduous screening of clones with desired features. This is especially important when the expression of the gene cannot be monitored as easily as a fluorescent protein. Additionally, by replacing the time-consuming G418 selection with that of Puro, we were able to shorten the time needed for obtaining Tet-On 3G-positive cell clones, a fundamental step in generation of inducible double stable cell lines, from more than two weeks to a few days. Puromycin-selected clones have been earlier shown to be extremely stable in maintenance of high transgene expression [30], which further supports the advantage of our redesigned system.

The adoption of the CAG promoter [19,31] instead of CMVp solves yet another source of problems with the original system, where the CMV promoter provides high gene expression but extended cell culturing and/or banking can result in its decreased activity over time [32,33] and transcriptional silencing associated with DNA methylation [34,35]. This is in line with our results, where the performance of stable CMV-based Tet-On-3G-cells after the extended cell line cultivation and banking was reduced drastically in contrast to our CAGp-based system providing durable, homogeneous, and high Tet-On 3G expression. By introducing the CAG promoter into the system, we were able to obtain a stable cell line with almost identical characteristics to that observed just after transactivator-positive-clone isolation, which has also been reported in other cell lines, even in demanding ones [20].

In the course of our present study, a similar bi-cistronic vector for co-expression of a transactivator and a selectable marker was successfully used to regulate embryonic stem cells and their differentiation into neuronal progenitors [36]. In such a system, the authors prepared one large lentiviral plasmid (12 kb) with EF-1 alpha promoter-driven expression of Puro and Tet-On 3G. The single plasmid also contained the GOI under the TRE3G promoter for one-step generation of the Dox-responding cell line. An advantage of such a vector is that lentiviral infection is extremely efficient, yet the efficient delivery of large vectors is only efficient via lentiviral transduction, which in turn requires additional time and a specialized safe unit for production thereof, which is not available in every laboratory or institution. Instead, our approach is based on a two-vector system, where it is possible to follow the standard procedure for developing double stable cell lines in a step-wise manner with two subsequent rounds of transfection and selection. Importantly, with this protocol, the transactivator-positive cell line once generated and characterized can be universally used to regulate the expression of any subsequent GOI. Additionally, we have demonstrated that our modification brings a possibility of a one-step ‘fast-track’ protocol based on co-transfection of both modified vectors at once and without the need of having advanced biosafety units. We have proved the functionality of this approach by expressing, in a tunable fashion, a gene of biological interest, the uL10 ribosomal protein fused with GFP. uL10 belongs to ribosomal proteins incorporated into the ribosomal particle late in the cytoplasmic stage of ribosome biogenesis, and perturbed balance of the uL10 expression exerts toxic effects [23]. Using the developed ‘fast-track’ protocol, we did not notice detectable leakage of the system in non-stimulated conditions, whereas a dose-dependent expression upon Dox was recorded. This kind of stringent regulation gives the opportunity to adjust the expression of GOIs with cellular state-specific expression to imitate its physiological or pathological influence on cells. We showed that the highly tunable system allowed the expression of uL10 in such a way that the majority of the hybrid uL10-GFP protein was incorporated into the ribosome. Moreover, we demonstrate that the initial features of the double stable cell line established with our system and protocol, effective and homogenous Dox responsiveness within cells, were preserved and stable even after the prolonged storage and cultivation.

Concluding, the new research tool based on the Tet-ON system and prepared with all the aforementioned modifications helps to establish a regulatory expression system in cell lines in a robust and efficient manner, with most of the desired features—tight expression control, high response, and homogeneity and stability in the cell.

## 4. Materials and Methods

### 4.1. Genetic Manipulations

The genetic construct pSpCas9(BB)-2A-Puro (PX459) V2.0, used as a backbone for construction of the regulatory pCAG-TET-T2A-Puro vector was kindly gifted by Feng Zhang (Addgene plasmid #62988; http://n2t.net/addgene:62988. accessed on 26 April 2018; RRID:Addgene_62988) [37]. The pCMV-TET3G vector used for amplification of the transactivator gene was purchased from Takara (Shiga, Japan). The uL10-GFP coding sequence for the response pTRE3G-EGFP-uL10-IRES vector was amplified by PCR on the pEGFP-C1-uL10 template described earlier [23] and cloned into the response pTRE3G-IRES vector, purchased from Takara, within the first MCS. The regulatory and response constructs were both prepared with a Gibson Assembly kit (New England BioLabs, Ipswitch, MA, USA), according to the manufacturer protocol. For the pTRE3G-GFP response vector, the GFP gene was PCR amplified from the pEGFP-C1 plasmid and inserted into pTRE3G-IRES in the first MCS by standard cloning with SalI and NdeI restriction enzymes (Thermo Fisher Scientific, Waltham, MA, USA). All PCR reactions were performed with a high-fidelity polymerase (KOD-Xtreme, Millipore, Burlington, MA, USA), following the instructions provided by the producer. The kanamycin/neomycin resistance cassette was amplified from the AmCyanP2AmCherry vector [38] and introduced into the response vectors by REPLACR-mutagenesis [21]. Each genetic construct prepared during the study was sequence-verified and is available in Addgene (plasmid #158067, #158068 and #158069) or can be obtained directly upon request to the corresponding author. All primers for cloning, mutagenesis, and sequencing were purchased from Genomed (Warsaw, Poland). They are presented in the Supplementary Section (Supplementary Table S1).

### 4.2. Cell Lines and Transfection

HEK293 and HeLa cells were purchased from ECACC (Porton Down, Salisbury, UK) and cultured in Ham’s DMEM/Nutrient Mixture F-12 supplemented with 10% fetal bovine serum, 100 IU/mL penicillin, and 100 μg/mL streptomycin. The MEF (DR-Wildtype) mouse embryonic fibroblasts and NIH3T3 mouse Swiss NIH embryo cell lines were purchased from ATCC (Manassas, VA, USA) and ECACC and grown in DMEM-high glucose and MEM, respectively, with 10% fetal bovine serum, 100 IU/mL penicillin, and 100 μg/mL streptomycin. All cell lines were cultured in a humid atmosphere of 5% CO_2_ at 37 °C and all aforementioned reagents were supplied by Merck Millipore (Burlington, MA, USA). The HEK293, HeLa, and NIH3T3 cells were transfected with DNA constructs using TurboFect transfection reagent (Thermo Fisher Scientific, Waltham, MA, USA) following the manufacturer instructions, whereas the MEF cells were transfected with GenJet™ In Vitro DNA Transfection Reagent (Ver. II) (SignaGen Laboratories, Rockville, MD, USA) according to the protocol provided by the supplier. The ratio of DNA/transfection reagent was optimized for each cell line in order to obtain the best transfection efficiency in the 24-well plate format.

### 4.3. Generation of Stable Cell Lines

The selection antibiotics, puromycin or geneticin, used for generation of single or double stable cell lines were purchased from Thermo Fisher Scientific, Gibco, (Waltham, MA, USA). The concentration of the antibiotics used for selection was determined by the kill curve prepared separately for each cell line. Antibiotic-resistant cell colonies were isolated with cloning cylinders (Merck). To this end, colony-forming cells surrounded by the cloning cylinder were washed with PBS and treated with trypsin-EDTA (Thermo Fisher Scientific, Waltham, MA, USA). After detachment, the cells were transferred with fresh growing medium supplemented with the selection antibiotics to a new well in a 24-well plate and grown to confluency. Colonies of antibiotic-resistant clonal cells with the best characteristics of transactivator expression (single stable cell lines) or Dox-induced GOI expression (double stable cell lines) were passaged into a larger flask, grown, and subjected to downstream analyses or frozen. For further cultivation of established stable cell lines a half the initial concentration of selection agents was used. Doxycycline used for induction of GOI expression was purchased from MP Biomedicals (Santa Ana, CA, USA). All cell lines established with the re-designed system and described in this work are available from the authors upon request.

### 4.4. Confocal Microscopy

Live-cell imaging was performed with the Laser Scanning Confocal Microscopy system LSM780 Zeiss (Oberkochen, Germany) built around an AxioObserverZ.1 inverted microscope equipped with a PlanApochromat 63x/1.40 Oil DIC M27 objective, two PMT (PhotoMultiplayerTube) detectors, a 32-channel GaAsP spectral detector, and an environmental chamber to control the CO_2_ concentration, air temperature, and humidity. The cells were grown in 35 ×10 mm glass bottom dishes (Greiner BioOne, Kremsmünster, Austria) and visualized after 48 h of Dox treatment. The analysis of the expression level and the steady-state subcellular localization of EGFP-hybrid proteins were carried out with a 488-nm laser line (2% of power) used for excitation and the PMT detector working at a 500–550 nm range. The pinhole diameter was set to 1 AU. For comparative studies of Dox-dose responsiveness, the gain in the GFP channel was the same and set each time to 790. Images and intensity profiles were acquired and prepared for reproduction with the use of ZEN2011 software (Carl Zeiss AG, Oberkochen, Germany).

### 4.5. Flow Cytometry of Living Cells

Transactivator-stable HEK293 cells transfected with the response plasmid and treated with Dox for 24 h were cultured on a 24-well plate Thermo Fisher Scientific, Nunc, (Waltham, MA, USA). The transfection efficiency was monitored under confocal microscopy to ensure that only cell populations with similarly equal transfection performance will be analyzed. Then, the cells were washed with Hank’s Balanced Salt Solution (HBSS, Thermo Fisher Scientific, Waltham, MA, USA) and detached from the plate by a Trypsin/EDTA solution (Merck Millipore, Burlington, MA, USA). Next, the cells were dissolved in 500 µL HBSS and analyzed on an FL-1 fluorescence channel using a FACSCalibur flow cytometer (BD, Franklin Lakes, NJ, USA). For each system, 20,000 cells were analyzed in triplicates with three biological replicates. The values of CV presented in the work were calculated with the CellQuest Pro software (version 6.0, BD, Franklin Lakes, NJ, USA) and were used to show the homogeneity of the cell populations. To this end, the estimation: CV = SD/mean was applied, where: SD is the standard deviation. Statistical analyses were performed using GraphPad Prism 5 (GraphPad Software, La Jolla, CA, USA). *p*-values ≤ 0.05 were considered to indicate statistical significance.

### 4.6. Polysome Profile Analysis and Immunoblotting

Polysome profiles were prepared from Dox-induced (50 ng/mL) DS HeLa cells after prolonged storage and cultivation. The cells were seeded at density 5x10^6^/15 cm dish and cultured overnight to obtain 75% confluency. Next, the cells were refreshed 3 h before harvesting by medium change and, after that time, treated with cycloheximide (CHX, 100 µg/mL) (Merck Millipore, Sigma-Aldrich, Saint Louis, MO, USA) for 5 min to block further protein synthesis. Then, the cells were washed 3 times with ice-cold TBS containing CHX (100 µg/mL), scraped off, and centrifuged at 2000× *g* for 5 min at 4 °C. The cell pellet was lysed in polysome lysis buffer (20 mM HEPES-KOH pH 7.4, 200 mM KCl, 5 mM MgCl_2_, 100 µg/mL CHX) supplemented with protease inhibitor cocktail (ProteaseSTOP) from Roche (Basel, Switzerland) and RNase Inhibitor (Merck Millipore, Sigma-Aldrich Saint Louis, MO, USA) and incubated on ice for 30 min with vortexing every 5 min. The cell lysate was additionally passed 15 times through a 23G needle, incubated on ice for 5 min with occasional mixing, and cleared by centrifugation at 10,000× *g* (rotor 12154-H; Sigma, Osterode am Harz, Germany) for 15 min at 4 °C. The RNA concentration was measured and 6 OD_260_ units of cell extract were loaded on a 15–50% sucrose gradient. The gradients were centrifuged in an SW32Ti rotor (Beckman-Coulter, Indianapolis, IN, USA) at 26,500 rpm for 4.5 h at 4 °C and then sampled using an ISCO Brandel (Gaithersburg, MD, USA) density gradient fractionator (BR-186). For immunodetection, proteins from each fraction were precipitated overnight with TCA at a final concentration of 10% and then washed three times with acetone. The dried pellet was resuspended in a 1xSDS-PAGE loading buffer and such samples were analyzed by Western blotting.

### 4.7. Cell Fractionation and Ribosome Purification

Dox-induced HeLa cells (50 ng/mL) stably expressing uL10-GFPC were fractionated and ribosomes were purified according to the standard procedure described in reference [39], with some modification. Briefly, the cells were washed 3 times with ice-cold TBS, harvested with a cell scraper, and centrifuged at 500× *g*, for 5 min at 4 °C. After disintegration in hypotonic buffer containing 0.5 μM of puromycin and 1% of IGEPAL CA-630, the cell homogenate was centrifuged at 800× *g* for 10 min at 4 °C to sediment the nuclei. Next, the cell extract was cleared by centrifugation (16 000× *g*, 15 min, 4 °C) to pellet mitochondria. The post-mitochondrial fraction was supplemented with KCl to the final concentration of 0.5 μM, layered onto a sucrose cushion containing 0.5 μM KCl, and centrifuged at 250,000× *g* for 3.5 h at 4 °C (rotor MLA-80; Beckman-Coulter, Brea, CA, USA) to purify and pellet ribosomes. The supernatant was collected and denoted as a post-ribosomal fraction. The post-nuclear cell extract and the ribosomal and post-ribosomal fractions were analyzed by Western blotting.

### 4.8. Immunodetection

Cell extracts of the HeLa double stable cell line subjected to various concentrations of Dox were prepared after 48 h of treatment with lysis buffer (Chromotek, Planegg-Martinsried, Germany) supplemented with the protease inhibitor cocktail, according to the manufacturer’s instructions. Cleared lysates with the same protein concentration were run on a 13.8% SDS-PAGE gel and proteins were transferred to a nitrocellulose membrane. Cellular and polysomal fractions were separated on 12% SDS-PAGE gel and transferred to the PVDF membrane. Western blotting analysis of the uL10-GFP fusion protein was performed with mouse monoclonal antibodies directed against GFP (Santa Cruz Biotechnology, Dallas, TX, USA). Loading controls were detected with the use of anti-GAPDH (Santa Cruz Biotechnology, Dallas, TX, USA) or anti-tubulin (Abcam, Cambridge, UK). Antibodies against uL10, uL16, and P1 proteins used in the analysis of cell and polysome fractions were acquired from Abcam (Cambridge, UK), Merck Millipore, Sigma-Aldrich (Saint Louis, MO, USA) and Atlas Antibodies (Bromma, Sweden), respectively. Immunoreactive bands were visualized with secondary antibodies conjugated to horseradish peroxidase (Bio-Rad, Hercules, CA, USA). Chemiluminescent blot signals were acquired using ChemiDoc™ MP (Bio-Rad, Hercules, CA, USA) and prepared for presentation with Image Lab 6.1 Software (Bio-Rad, Hercules, CA, USA).

## Figures and Tables

**Table 1:** Comparison of Tet-On 3G and Tet-On 3G-T2A-Puro inducible expression systems.

Feature	Tet-On 3G	Tet-On 3G-T2A-Puro	Advantages of New System Tet-On 3G-T2A-Puro
REGULATORY PLASMID			
Promotor	CMV	CAG	Less prone to silencing through methylationPersistent expression of transactivator
Selection cassette	Kan/Neo	Puro	Fast selection of resistant clones
Days of selection	>14	4–8	
Co-expression of selection cassette and transactivator	−	+	Every resistant clonal cell expresses transactivator (higher efficiency)
RESPONSE PLASMID			
Selection cassette	external (linear for co-transfection)	internal (within plasmid backbone)	Double stable cell line establishment with regulated GOI expression according to ‘fast-track’ protocol
Procedure	two-step	two-step or one-step	Alternative protocols possible: standard or rapid ‘fast-track’

## Supplementary Materials

The following are available online at https://www.mdpi.com/article/10.3390/ijms22105206/s1.

## References

[1] Gossen M., Bujard H. (1992). Tight control of gene expression in mammalian cells by tetracycline-responsive promoters. Proc. Natl. Acad. Sci. USA.

[2] Mullick A., Xu Y., Warren R., Koutroumanis M., Guilbault C., Broussau S., Malenfant F., Bourget L., Lamoureux L., Lo R. (2006). The cumate gene-switch: A system for regulated expression in mammalian cells. BMC Biotechnol..

[3] Rivera V.M., Clackson T., Natesan S., Pollock R., Amara J.F., Keenan T., Magari S.R., Phillips T., Courage N.L., Cerasoli F. (1996). A humanized system for pharmacologic control of gene expression. Nat. Med..

[4] Zhang Y., Riesterer C., Ayrall A.M., Sablitzky F., Littlewood T.D., Reth M. (1996). Inducible site-directed recombination in mouse embryonic stem cells. Nucleic Acids Res..

[5] Winkler W.C., Nahvi A., Roth A., Collins J.A., Breaker R.R. (2004). Control of gene expression by a natural metabolite-responsive ribozyme. Nature.

[6] Kallunki T., Barisic M., Jaattela M., Liu B. (2019). How to Choose the Right Inducible Gene Expression System for Mammalian Studies?. Cells.

[7] Das A.T., Tenenbaum L., Berkhout B. (2016). Tet-On Systems For Doxycycline-inducible Gene Expression. Curr. Gene Ther..

[8] Choi K.H., Basma H., Singh J., Cheng P.W. (2005). Activation of CMV promoter-controlled glycosyltransferase and beta -galactosidase glycogenes by butyrate, tricostatin A, and 5-aza-2′-deoxycytidine. Glycoconj J..

[9] Krishnan M., Park J.M., Cao F., Wang D., Paulmurugan R., Tseng J.R., Gonzalgo M.L., Gambhir S.S., Wu J.C. (2006). Effects of epigenetic modulation on reporter gene expression: Implications for stem cell imaging. FASEB J..

[10] Ban N., Beckmann R., Cate J.H., Dinman J.D., Dragon F., Ellis S.R., Lafontaine D.L., Lindahl L., Liljas A., Lipton J.M. (2014). A new system for naming ribosomal proteins. Curr. Opin. Struct. Biol..

[11] Derylo K., Michalec-Wawiorka B., Krokowski D., Wawiorka L., Hatzoglou M., Tchorzewski M. (2018). The uL10 protein, a component of the ribosomal P-stalk, is released from the ribosome in nucleolar stress. Biochim. Biophys. Acta Mol. Cell Res..

[12] Filipek K., Michalec-Wawiorka B., Boguszewska A., Kmiecik S., Tchorzewski M. (2020). Phosphorylation of the N-terminal domain of ribosomal P-stalk protein uL10 governs its association with the ribosome. FEBS Lett..

[13] Barnard G.F., Staniunas R.J., Bao S., Mafune K., Steele G.D., Gollan J.L., Chen L.B. (1992). Increased expression of human ribosomal phosphoprotein P0 messenger RNA in hepatocellular carcinoma and colon carcinoma. Cancer Res..

[14] Kondoh N., Wakatsuki T., Ryo A., Hada A., Aihara T., Horiuchi S., Goseki N., Matsubara O., Takenaka K., Shichita M. (1999). Identification and characterization of genes associated with human hepatocellular carcinogenesis. Cancer Res..

[15] Lai M.D., Xu J. (2007). Ribosomal proteins and colorectal cancer. Curr. Genom..

[16] Chang T.W., Chen C.C., Chen K.Y., Su J.H., Chang J.H., Chang M.C. (2008). Ribosomal phosphoprotein P0 interacts with GCIP and overexpression of P0 is associated with cellular proliferation in breast and liver carcinoma cells. Oncogene.

[17] Artero-Castro A., Castellvi J., Garcia A., Hernandez J., Cajal S.R., Lleonart M.E. (2011). Expression of the ribosomal proteins Rplp0, Rplp1, and Rplp2 in gynecologic tumors. Hum. Pathol..

[18] Teller A., Jechorek D., Hartig R., Adolf D., Reissig K., Roessner A., Franke S. (2015). Dysregulation of apoptotic signaling pathways by interaction of RPLP0 and cathepsin X/Z in gastric cancer. Pathol. Res. Pract..

[19] Hitoshi N., Ken-ichi Y., Jun-ichi M. (1991). Efficient selection for high-expression transfectants with a novel eukaryotic vector. Gene.

[20] Alexopoulou A.N., Couchman J.R., Whiteford J.R. (2008). The CMV early enhancer/chicken beta actin (CAG) promoter can be used to drive transgene expression during the differentiation of murine embryonic stem cells into vascular progenitors. BMC Cell Biol..

[21] Trehan A., Kielbus M., Czapinski J., Stepulak A., Huhtaniemi I., Rivero-Muller A. (2016). REPLACR-mutagenesis, a one-step method for site-directed mutagenesis by recombineering. Sci. Rep..

[22] Tchorzewski M., Krokowski D., Rzeski W., Issinger O.G., Grankowski N. (2003). The subcellular distribution of the human ribosomal “stalk” components: P1, P2 and P0 proteins. Int. J. Biochem. Cell Biol..

[23] Michalec B., Krokowski D., Grela P., Wawiorka L., Sawa-Makarska J., Grankowski N., Tchorzewski M. (2010). Subcellular localization of ribosomal P0-like protein MRT4 is determined by its N-terminal domain. Int. J. Biochem. Cell Biol..

[24] Gossen M., Freundlieb S., Bender G., Muller G., Hillen W., Bujard H. (1995). Transcriptional activation by tetracyclines in mammalian cells. Science.

[25] Loew R., Heinz N., Hampf M., Bujard H., Gossen M. (2010). Improved Tet-responsive promoters with minimized background expression. BMC Biotechnol..

[26] Urlinger S., Baron U., Thellmann M., Hasan M.T., Bujard H., Hillen W. (2000). Exploring the sequence space for tetracycline-dependent transcriptional activators: Novel mutations yield expanded range and sensitivity. Proc. Natl. Acad. Sci. USA.

[27] Zhou X., Vink M., Klaver B., Berkhout B., Das A.T. (2006). Optimization of the Tet-On system for regulated gene expression through viral evolution. Gene Ther..

[28] Szymczak-Workman A.L., Vignali K.M., Vignali D.A. (2012). Design and construction of 2A peptide-linked multicistronic vectors. Cold Spring Harb. Protoc..

[29] Park K., Jeong J., Chung B.H. (2014). Live imaging of cellular dynamics using a multi-imaging vector in single cells. Chem. Commun..

[30] Lanza A.M., Kim D.S., Alper H.S. (2013). Evaluating the influence of selection markers on obtaining selected pools and stable cell lines in human cells. Biotechnol. J..

[31] Miyazaki J., Takaki S., Araki K., Tashiro F., Tominaga A., Takatsu K., Yamamura K. (1989). Expression vector system based on the chicken beta-actin promoter directs efficient production of interleukin-5. Gene.

[32] Bailey L.A., Hatton D., Field R., Dickson A.J. (2012). Determination of Chinese hamster ovary cell line stability and recombinant antibody expression during long-term culture. Biotechnol. Bioeng..

[33] He L., Winterrowd C., Kadura I., Frye C. (2012). Transgene copy number distribution profiles in recombinant CHO cell lines revealed by single cell analyses. Biotechnol. Bioeng..

[34] Hsu C.C., Li H.P., Hung Y.H., Leu Y.W., Wu W.H., Wang F.S., Lee K.D., Chang P.J., Wu C.S., Lu Y.J. (2010). Targeted methylation of CMV and E1A viral promoters. Biochem. Biophys. Res. Commun..

[35] Moritz B., Becker P.B., Gopfert U. (2015). CMV promoter mutants with a reduced propensity to productivity loss in CHO cells. Sci. Rep..

[36] Liu Y., Yu C., Daley T.P., Wang F., Cao W.S., Bhate S., Lin X., Still I.I.C., Liu H., Zhao D. (2018). CRISPR Activation Screens Systematically Identify Factors that Drive Neuronal Fate and Reprogramming. Cell Stem Cell.

[37] Ran F.A., Hsu P.D., Wright J., Agarwala V., Scott D.A., Zhang F. (2013). Genome engineering using the CRISPR-Cas9 system. Nat. Protoc..

[38] Potorac I., Rivero-Muller A., Trehan A., Kielbus M., Jozwiak K., Pralong F., Hafidi A., Thiry A., Ménagé J.J., Huhtaniemi I.T. (2016). A vital region for human glycoprotein hormone trafficking revealed by an LHB mutation. J. Endocrinol..

[39] Belin S., Hacot S., Daudignon L., Therizols G., Pourpe S., Mertani H.C., Rosa-Calatrava M., Diaz J.J. (2010). Purification of ribosomes from human cell lines. Curr. Protoc. Cell Biol..

