# Supplementary material for: An Improved Vector System for Homogeneous and Stable Gene Regulation
Source: Int J Mol Sci. 2021 May 14;22(10):5206. doi: 10.3390/ijms22105206 (PMC8157167; doi:10.3390/ijms22105206)
Supplement: Supplementary file 1 [file ijms-22-05206-s001.zip › ijms-1184172-supplementary.pdf]

Supplementary information

An improved vector system for homogeneous and stable gene regulation

Barbara Michalec-Wawiórka[1]*, Jakub Czapiński[2,3], Kamil Filipek[1], Patrycja Rulak[1], Arkadiusz Czerwonka[2], Marek Tchórzewski[1], Adolfo Rivero-Müller[2]

[1] Department of Molecular Biology, Institute of Biological Sciences, Maria Curie-Skłodowska University, 20-033 Lublin, Poland

[2] Department of Biochemistry and Molecular Biology, Medical University of Lublin, 20-093 Lublin, Poland

[3] Postgraduate School of Molecular Medicine, 02-091Warsaw, Poland

* Correspondence should be addressed to Barbara Michalec-Wawiórka: basiam@hektor.umcs.lublin.pl

Figures:

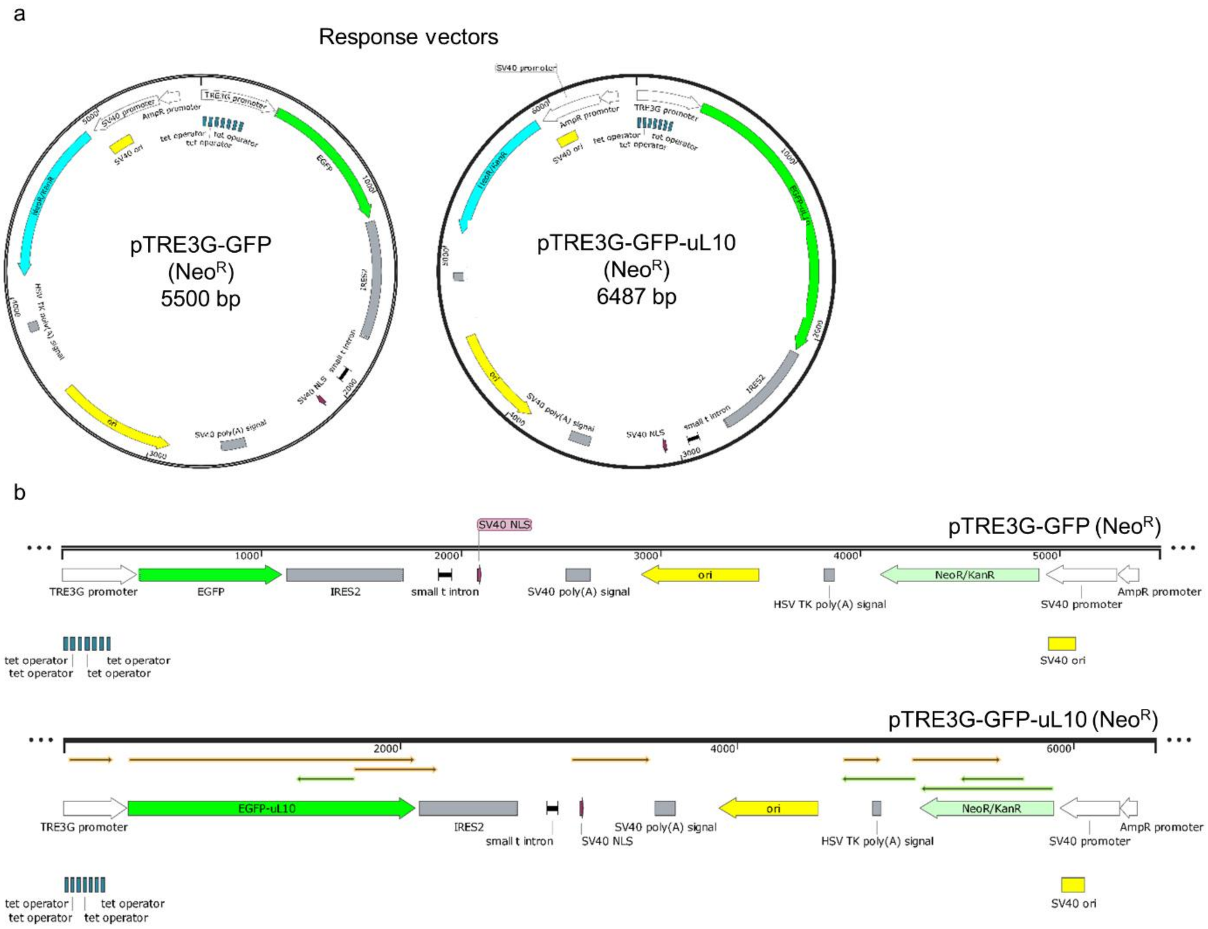

Figure S1. Genetic maps of response vectors prepared for Tet-On 3G-T2A-Puro system validation. Both response vectors pTRE3G-GFP and pTRE3G-GFP-uL10 presented in circular (a) and linear (b) views have the *Kan/Neo* selectable marker and contain the reporter gene *EGFP* or the gene of biological interest coding human uL10 protein fused with GFP, respectively, under the Tet-O promoter. The vector maps were prepared with SnapGene (SnapGene® software, from GSL Biotech; available at snapgene.com).

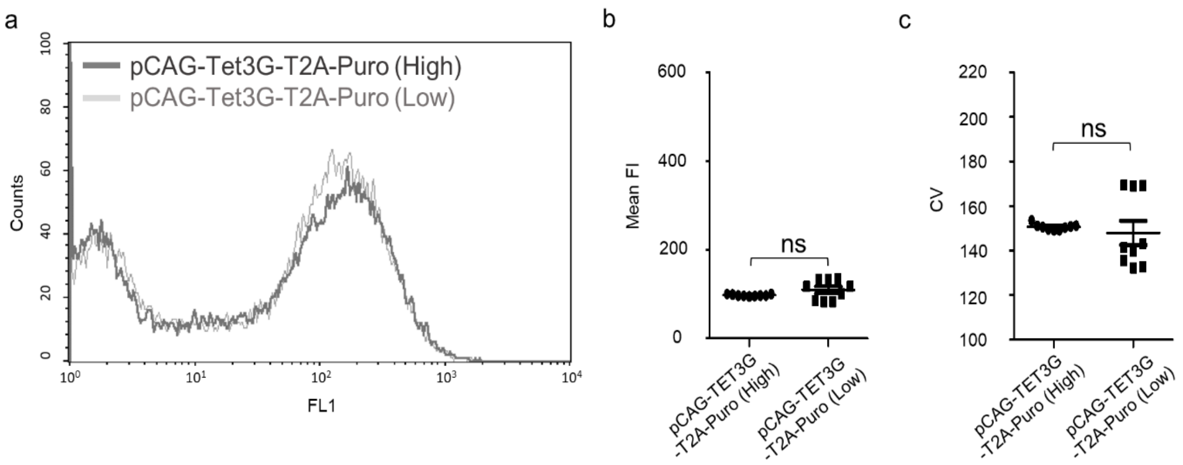

Figure S2. Comparison of GFP expression in pCAG-TET3G-T2A-Puro-cells selected with different concentrations of puromycin. The parameters of the GFP transgene expression were measured by flow cytometry in inducible HEK293 (Human Embryo Kidney) cell lines generated with the TetOn-3G-T2A-Puro system and selected with high (1.2 µg/ml) or low (0.5 µg/ml) puromycin (Puro) concentrations. The stable cell lines show similar mean fluorescence intensity of the GFP transgene and the profile of its expression independently of the Puro concentration used for selection, which is displayed in flow cytometry histograms (a) and reflected in mean fluorescence intensity (FI) (b) and coefficient of variation (CV) measurements (c) presented in plots. Statistical significance of differences between both stable cell lines was calculated using the Mann-Whitney test; ns – not significant.

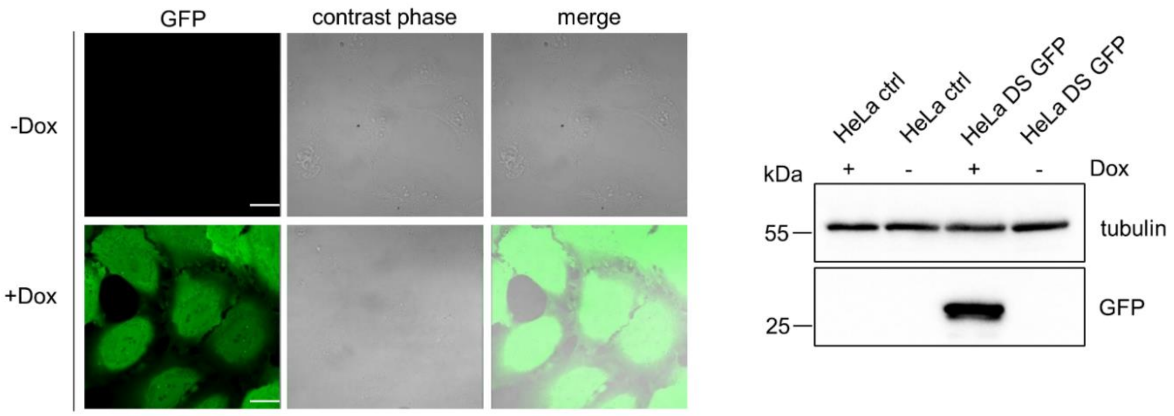

Figure S3. Dox-induced expression of the GFP reporter in a double-stable HeLa cell line established according to the standard two-step protocol. The HeLa cell line stably expressing the transactivator (with integrated pCAG-TET3G-T2A-Puro) was transfected with the pTRE3G-GFP response plasmid and, after 24h, treated with doxycycline (Dox, 500 ng/ml) to monitor induced expression of GFP. Next, geneticin (G418, 1200 µg/ml) was applied and, after 14 days of selection, individual clones were isolated and analyzed for Dox-induced expression with confocal microscopy and western blotting. *Left panel:* Live-cell images of the double stable cell line treated (+Dox) or untreated (-Dox) with doxycycline. Green fluorescence of GFP is shown on the left, the contrast phase view of the cells is shown in the middle panel, and merged images are presented on the right; scale bar 20 µm. *Right panel*: Immunodetection of GFP expression in response to Dox induction. Control HeLa cells (HeLa ctrl) and double-stable HeLa cells with inducible GFP expression (HeLa DS GFP) were treated with doxycycline (+Dox) or left untreated (-Dox), lysed, and analyzed by Western blot with anti-GFP antibody. Antibodies against tubulin were used as a loading control. Full-length blots are presented in Fig. S3.

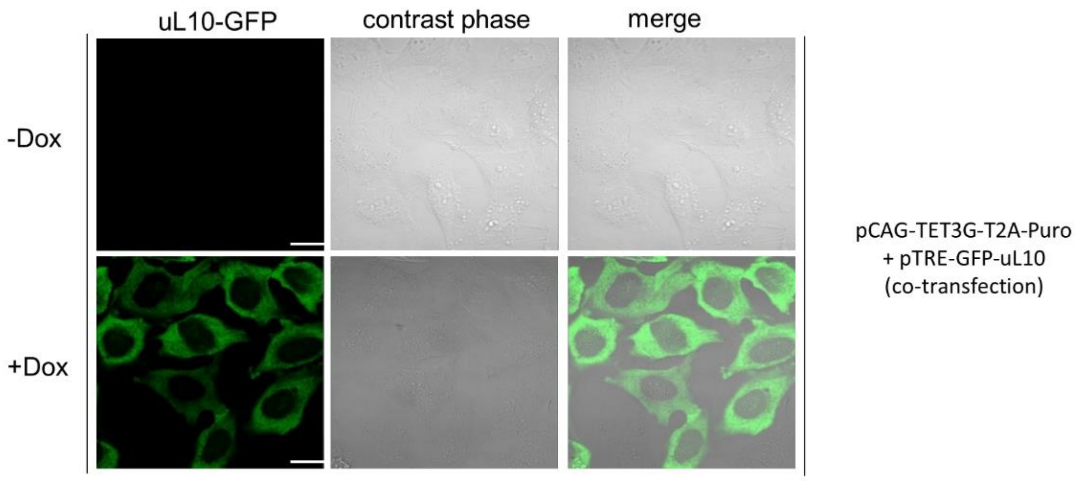

Figure S4. Functionality test of the optimized Tet-On 3G-T2A-Puro system used for uL10-GFP regulated expression. HeLa cells were co-transfected with regulatory pCAG-TET3G-T2A-Puro and response pTRE3G-GFP-uL10 plasmids. 24h after transfection, Dox (500 ng/ml) was administered, and induced expression of uL10-GFP was monitored with confocal microscopy after 24h. Live-cell images of transfected cells treated (+Dox) or untreated (-Dox) with doxycycline are presented. Fluorescence of uL10-GFP is shown on the left, the contrast phase view of the cells is shown in the middle panel, and merged images are presented on the right; scale bar 20 µm.

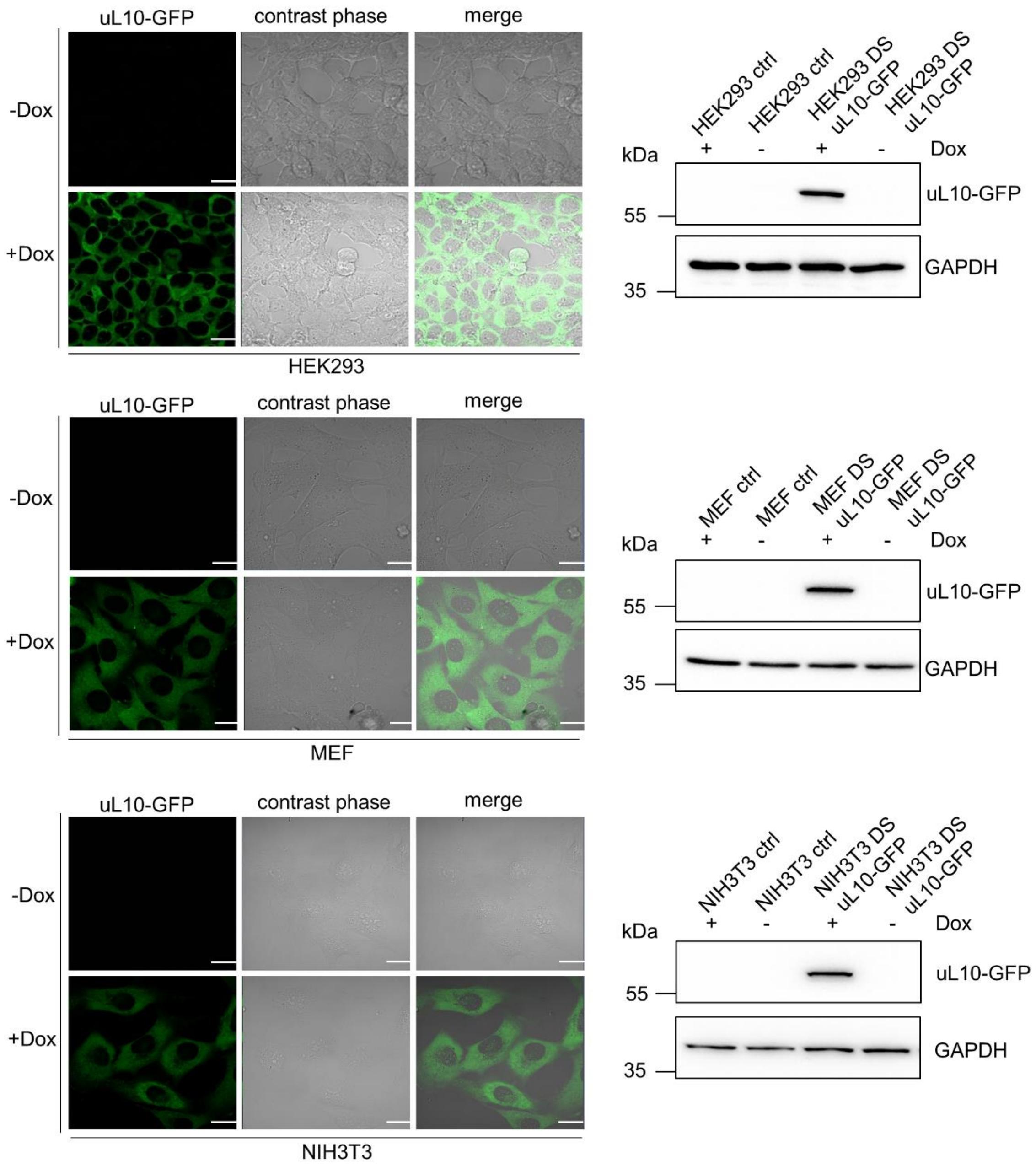

Figure S5. Dox-induced expression of the uL10-GFP protein in double-stable cell lines: HEK293, MEF (mouse embryonic fibroblasts), and NIH3T3 (mouse Swiss NIH embryonic fibroblasts). Cell lines expressing the uL10-GFP fusion protein in response to doxycycline treatment were established according to the 'fast-track' protocol. *Left panel:* Live-cell images of double stable cells treated with doxycycline (+Dox) or untreated (-Dox). Hybrid uL10-GFP is shown on the left, the contrast phase view of cells is shown in the middle, and a merged image of two previous pictures is presented on the right; scale bar 20 µm. *Right panel*: Immunodetection of the uL10-GFP protein expression in response to Dox induction. Control cells (HEK293 ctrl; MEF ctrl; NIH3T3 ctrl) and double-stable cells with inducible uL10-GFP expression (HEK293 DS uL10-GFP; MEF DS uL10-GFP; NIH3T3 DS uL10-GFP) were

treated with doxycycline (+Dox) or left untreated (-Dox), lysed, and analyzed by Western blot with anti-GFP antibody. Antibodies against GAPDH protein were used as a loading control. Full-length blots are presented in Figure S8.

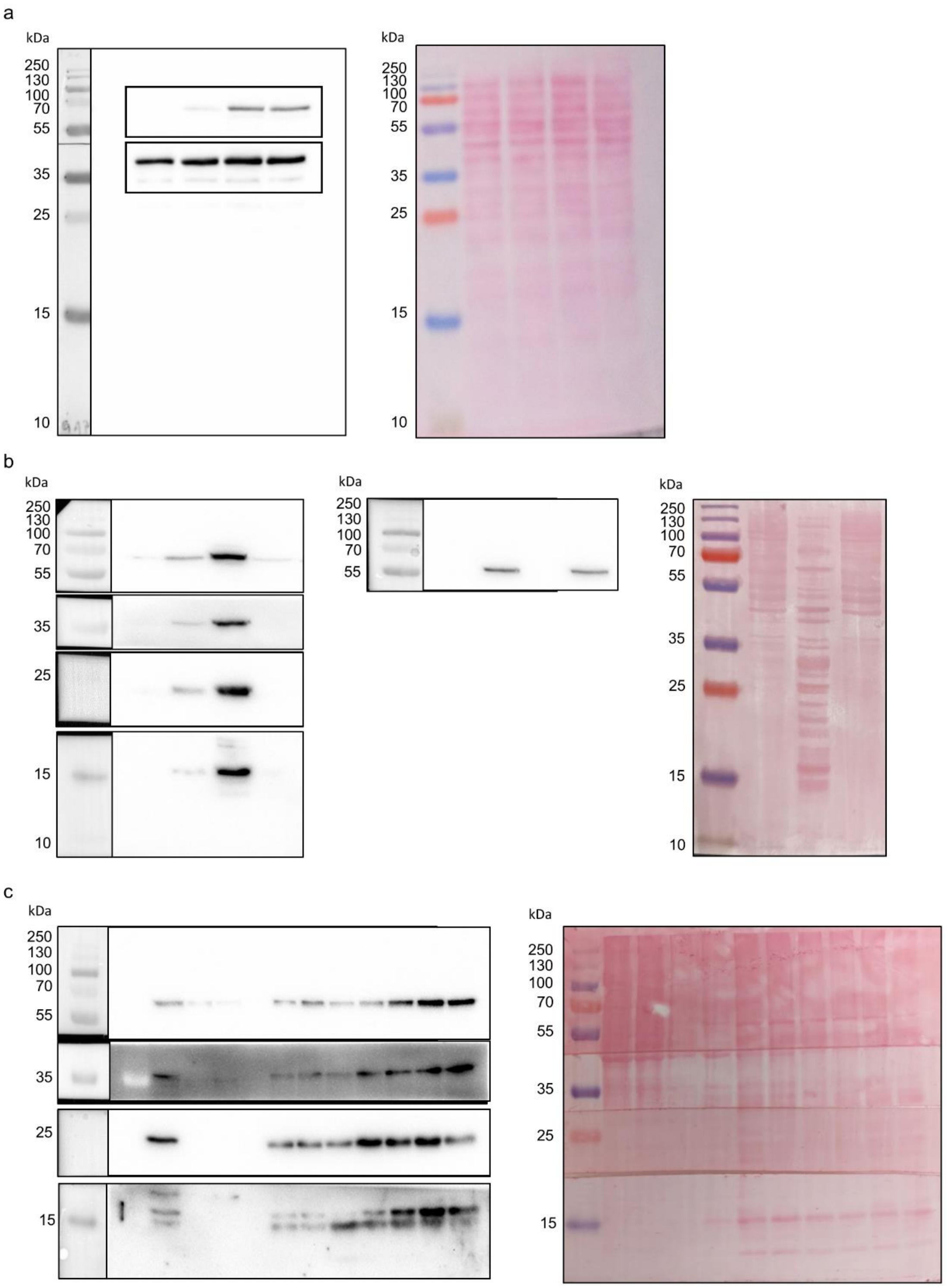

Figure S6. Full-length blots with protein markers and nitrocellulose membranes stained with Ponceau S from Figure 5 showing: (**a**) protein loading in cell extract samples from DS HeLa cells treated with different concentrations of Dox; (**b**) protein loading and composition of cellular fractions from Dox-induced DS HeLa cells; (**c**) protein loading and composition of polysome profile fractions from Dox-induced DS HeLa cells. Membranes were cut before

development with particular antibodies to provide robust analysis of exactly the same sample with every antibodies used. All membrane parts were developed and acquired separately.

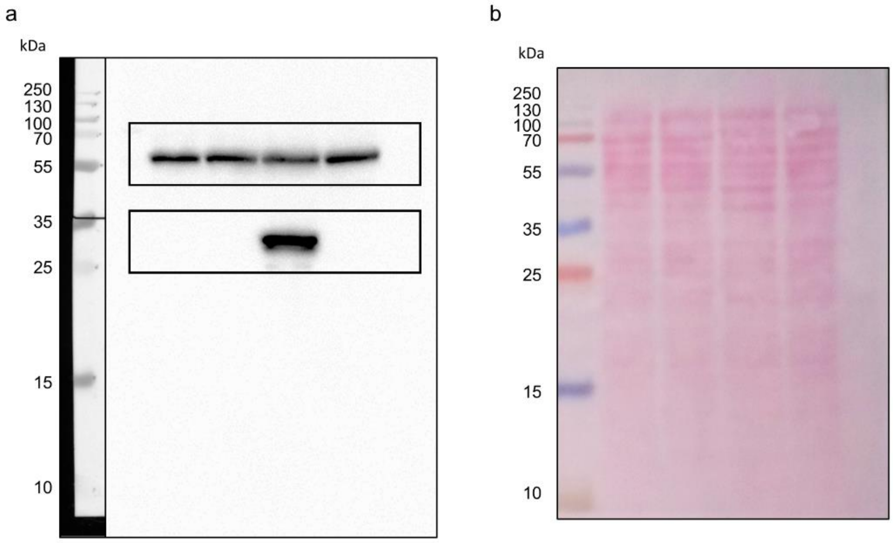

Figure S7. Full-length blot with protein markers (**a**) and a nitrocellulose membrane stained with Ponceau S showing protein loading in cell extract samples (**b**) from Fig. S3. Membrane was cut before development with particular antibodies to provide robust analysis of exactly the same sample with every antibodies used.

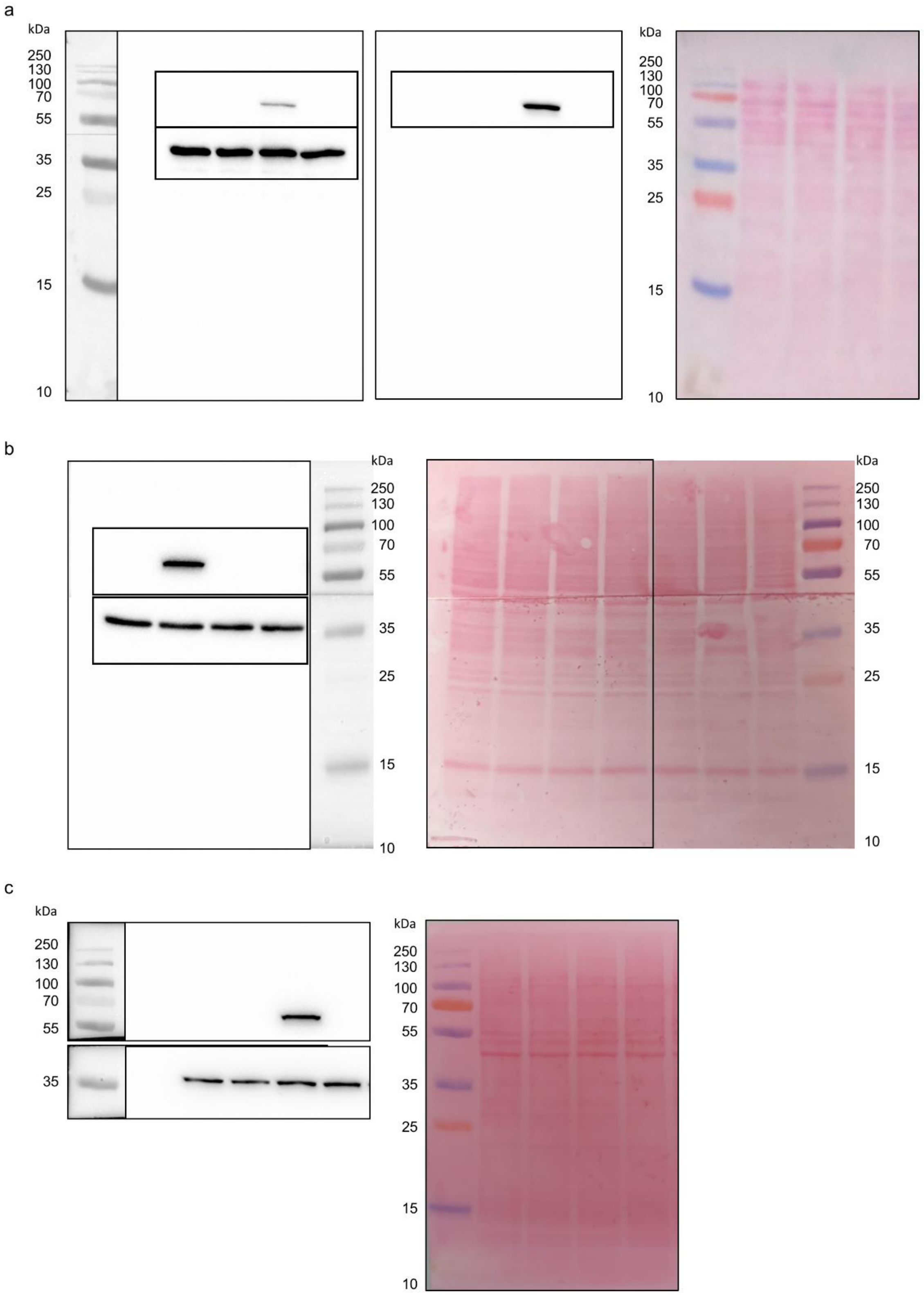

Figure S8. Full-length blots with protein markers and nitrocellulose membranes stained with Ponceau S showing protein loading in cell extract samples from double stable cell lines: HEK293 (**a**), where parallel development of GFP and GAPDH antibodies signals (*left panel*) and GFP signal development only (*middle panel*) is shown; MEF (**b**), with a black frame

indicating the analyzed region of the membrane; NIH3T3 (**c**), presented in Figure S5. Membranes were cut before development with particular antibodies to provide robust analysis of exactly the same sample with every antibodies used.

Genetic construct sequences:

pCAG-TET3G-T2A-Puro

GAGGGCCTATTTCCCATGATTCCTTCATATTTGCATATACGATACAAGGCTGTTAGAGAGATAATTG
GAATTAATTTGACTGTAAACACAAAGATATTAGTACAAAATACGTGACGTAGAAAGTAATAATTTC
TTGGGTAGTTTGCAGTTTTAAAATTATGTTTTAAAATGGACTATCATATGCTTACCGTAACTTGAAA
GTATTTCGATTTCTTGGCTTTATATATCTTGTGGAAAGGACGAAACACCGGGTCTTCGAGAAGACCT
GTTTTAGAGCTAGAAATAGCAAGTTAAAATAAGGCTAGTCCGTTATCAACTTGAAAAAGTGGCACC
GAGTCGGTGCTTTTTTGTTTTAGAGCTAGAAATAGCAAGTTAAAATAAGGCTAGTCCGTTTTTAGCG
CGTGCGCCAATTCTGCAGACAAATGGCTCTAGAGGTACCCGTTACATAACTTACGGTAAATGGCCC
GCCTGGCTGACCGCCCAACGACCCCCGCCCATTGACGTCAATAGTAACGCCAATAGGGACTTTCCA
TTGACGTCAATGGGTGGAGTATTTACGGTAAACTGCCCACTTGGCAGTACATCAAGTGTATCATAT
GCCAAGTACGCCCCCTATTGACGTCAATGACGGTAAATGGCCCGCCTGGCATTGTGCCCAGTACAT
GACCTTATGGGACTTTCCTACTTGGCAGTACATCTACGTATTAGTCATCGCTATTACCATGGTCGAG
GTGAGCCCCACGTTCTGCTTCACTCTCCCCATCTCCCCCCCCTCCCCACCCCCAATTTTGTATTTATT
TATTTTTTAATTATTTTGTGCAGCGATGGGGGCGGGGGGGGGGGGGGGGCGCGCGCCAGGCGGGG
CGGGGCGGGGCGAGGGGCGGGGCGGGGCGAGGCGGAGAGGTGCGGCGGCAGCCAATCAGAGCGG
CGCGCTCCGAAAGTTTCCTTTTATGGCGAGGCGGCGGCGGCGGCGGCCCTATAAAAAGCGAAGCG
CGCGGCGGGCGGGAGTCGCTGCGACGCTGCCTTCGCCCCGTGCCCCGCTCCGCCGCCGCCTCGCGC
CGCCCGCCCCGGCTCTGACTGACCGCGTTACTCCCACAGGTGAGCGGGCGGGACGGCCCTTCTCCT
CCGGGCTGTAATTAGCTGAGCAAGAGGTAAGGGTTTAAGGGATGGTTGGTTGGTGGGGTATTAATG
TTTAATTACCTGGAGCACCTGCCTGAAATCACTTTTTTTCAGGTTGGACCGGTGCCACCATGTCTAG
ACTGGACAAGAGCAAAGTCATAAACTCTGCTCTGGAATTACTCAATGGAGTCGGTATCGAAGGCCT
GACGACAAGGAAACTCGCTCAAAAGCTGGGAGTTGAGCAGCCTACCCTGTACTGGCACGTGAAGA
ACAAGCGGGCCCTGCTCGATGCCCTGCCAATCGAGATGCTGGACAGGCATCATACCCACTCCTGCC
CCCTGGAAGGCGAGTCATGGCAAGACTTTCTGCGGAACAACGCCAAGTCATACCGCTGTGCTCTCC
TCTCACATCGCGACGGGGCTAAAGTGCATCTCGGCACCCGCCCAACAGAGAAACAGTACGAAACC
CTGGAAAATCAGCTCGCGTTCCTGTGTCAGCAAGGCTTCTCCCTGGAGAACGCACTGTACGCTCTG
TCCGCCGTGGGCCACTTTACACTGGGCTGCGTATTGGAGGAACAGGAGCATCAAGTAGCAAAAGA
GGAAAGAGAGACACCTACCACCGATTCTATGCCCCCACTTCTGAAACAAGCAATTGAGCTGTTCGA
CCGGCAGGGAGCCGAACCTGCCTTCCTTTTCGGCCTGGAACTAATCATATGTGGCCTGGAGAAACA
GCTAAAGTGCGAAAGCGGCGGGCCGACCGACGCCCTTGACGATTTTGACTTAGACATGCTCCCAGC
CGATGCCCTTGACGACTTTGACCTTGATATGCTGCCTGCTGACGCTCTTGACGATTTTGACCTTGAC
ATGCTCCCCGGGGAATTCGGCAGTGGAGAGGGCAGAGGAAGTCTGCTAACATGCGGTGACGTCGA
GGAGAATCCTGGCCCAATGACCGAGTACAAGCCCACGGTGCGCCTCGCCACCCGCGACGACGTCC
CCAGGGCCGTACGCACCCTCGCCGCCGCGTTCGCCGACTACCCCGCCACGCGCCACACCGTCGATC
CGGACCGCCACATCGAGCGGGTCACCGAGCTGCAAGAACTCTTCCTCACGCGCGTCGGGCTCGACA
TCGGCAAGGTGTGGGTCGCGGACGACGGCGCCGCGGTGGCGGTCTGGACCACGCCGGAGAGCGTC
GAAGCGGGGGCGGTGTTCGCCGAGATCGGCCCGCGCATGGCCGAGTTGAGCGGTTCCCGGCTGGC
CGCGCAGCAACAGATGGAAGGCCTCCTGGCGCCGCACCGGCCCAAGGAGCCCGCGTGGTTCCTGG
CCACCGTCGGAGTCTCGCCCGACCACCAGGGCAAGGGTCTGGGCAGCGCCGTCGTGCTCCCCGGAG
TGGAGGCGGCCGAGCGCGCCGGGGTGCCCGCCTTCCTGGAGACCTCCGCGCCCCGCAACCTCCCCT
TCTACGAGCGGCTCGGCTTCACCGTCACCGCCGACGTCGAGGTGCCCGAAGGACCGCGCACCTGGT
GCATGACCCGCAAGCCCGGTGCCTGAGAATTCTAACTAGAGCTCGCTGATCAGCCTCGACTGTGCC

TTCTAGTTGCCAGCCATCTGTTGTTTGCCCCTCCCCCGTGCCTTCCTTGACCCTGGAAGGTGCCACTC
CCACTGTCCTTTCCTAATAAAATGAGGAAATTGCATCGCATTGTCTGAGTAGGTGTCATTCTATTCT
GGGGGGTGGGGTGGGGCAGGACAGCAAGGGGGAGGATTGGGAAGAGAATAGCAGGCATGCTGGG
GAGCGGCCGCAGGAACCCCTAGTGATGGAGTTGGCCACTCCCTCTCTGCGCGCTCGCTCGCTCACT
GAGGCCGGGCGACCAAAGGTCGCCCGACGCCCGGGCTTTGCCCGGGCGGCCTCAGTGAGCGAGCG
AGCGCGCAGCTGCCTGCAGGGGCGCCTGATGCGGTATTTTCTCCTTACGCATCTGTGCGGTATTTCA
CACCGCATACGTCAAAGCAACCATAGTACGCGCCCTGTAGCGGCGCATTAAGCGCGGCGGGTGTG
GTGGTTACGCGCAGCGTGACCGCTACACTTGCCAGCGCCCTAGCGCCCGCTCCTTTCGCTTTCTTCC
CTTCCTTTCTCGCCACGTTCGCCGGCTTTCCCCGTCAAGCTCTAAATCGGGGGCTCCCTTTAGGGTT
CCGATTTAGTGCTTTACGGCACCTCGACCCCAAAAAACTTGATTTGGGTGATGGTTCACGTAGTGG
GCCATCGCCCTGATAGACGGTTTTTCGCCCTTTGACGTTGGAGTCCACGTTCTTTAATAGTGGACTC
TTGTTCCAAACTGGAACAACACTCAACCCTATCTCGGGCTATTCTTTTGATTTATAAGGGATTTTGC
CGATTTCGGCCTATTGGTTAAAAAATGAGCTGATTTAACAAAAATTTAACGCGAATTTTAACAAAA
TATTAACGTTTACAATTTTATGGTGCACTCTCAGTACAATCTGCTCTGATGCCGCATAGTTAAGCCA
GCCCCGACACCCGCCAACACCCGCTGACGCGCCCTGACGGGCTTGTCTGCTCCCGGCATCCGCTTA
CAGACAAGCTGTGACCGTCTCCGGGAGCTGCATGTGTCAGAGGTTTTCACCGTCATCACCGAAACG
CGCGAGACGAAAGGGCCTCGTGATACGCCTATTTTTATAGGTTAATGTCATGATAATAATGGTTTCT
TAGACGTCAGGTGGCACTTTTCGGGGAAATGTGCGCGGAACCCCTATTTGTTTATTTTTCTAAATAC
ATTCAAATATGTATCCGCTCATGAGACAATAACCCTGATAAATGCTTCAATAATATTGAAAAAGGA
AGAGTATGAGTATTCAACATTTCCGTGTCGCCCTTATTCCCTTTTTTGCGGCATTTTGCCTTCCTGTT
TTTGCTCACCCAGAAACGCTGGTGAAAGTAAAAGATGCTGAAGATCAGTTGGGTGCACGAGTGGG
TTACATCGAACTGGATCTCAACAGCGGTAAGATCCTTGAGAGTTTTCGCCCCGAAGAACGTTTTCC
AATGATGAGCACTTTTAAAGTTCTGCTATGTGGCGCGGTATTATCCCGTATTGACGCCGGGCAAGA
GCAACTCGGTCGCCGCATACACTATTCTCAGAATGACTTGGTTGAGTACTCACCAGTCACAGAAAA
GCATCTTACGGATGGCATGACAGTAAGAGAATTATGCAGTGCTGCCATAACCATGAGTGATAACAC
TGCGGCCAACTTACTTCTGACAACGATCGGAGGACCGAAGGAGCTAACCGCTTTTTTGCACAACAT
GGGGGATCATGTAACTCGCCTTGATCGTTGGGAACCGGAGCTGAATGAAGCCATACCAAACGACG
AGCGTGACACCACGATGCCTGTAGCAATGGCAACAACGTTGCGCAAACTATTAACTGGCGAACTAC
TTACTCTAGCTTCCCGGCAACAATTAATAGACTGGATGGAGGCGGATAAAGTTGCAGGACCACTTC
TGCGCTCGGCCCTTCCGGCTGGCTGGTTTATTGCTGATAAATCTGGAGCCGGTGAGCGTGGAAGCC
GCGGTATCATTGCAGCACTGGGGCCAGATGGTAAGCCCTCCCGTATCGTAGTTATCTACACGACGG
GGAGTCAGGCAACTATGGATGAACGAAATAGACAGATCGCTGAGATAGGTGCCTCACTGATTAAG
CATTGGTAACTGTCAGACCAAGTTTACTCATATATACTTTAGATTGATTTAAAACTTCATTTTTAATT
TAAAAGGATCTAGGTGAAGATCCTTTTTGATAATCTCATGACCAAAATCCCTTAACGTGAGTTTTCG
TTCCACTGAGCGTCAGACCCCGTAGAAAAGATCAAAGGATCTTCTTGAGATCCTTTTTTTCTGCGCG
TAATCTGCTGCTTGCAAACAAAAAAACCACCGCTACCAGCGGTGGTTTGTTTGCCGGATCAAGAGC
TACCAACTCTTTTTCCGAAGGTAACTGGCTTCAGCAGAGCGCAGATACCAAATACTGTCCTTCTAGT
GTAGCCGTAGTTAGGCCACCACTTCAAGAACTCTGTAGCACCGCCTACATACCTCGCTCTGCTAATC
CTGTTACCAGTGGCTGCTGCCAGTGGCGATAAGTCGTGTCTTACCGGGTTGGACTCAAGACGATAG
TTACCGGATAAGGCGCAGCGGTCGGGCTGAACGGGGGGTTCGTGCACACAGCCCAGCTTGGAGCG
AACGACCTACACCGAACTGAGATACCTACAGCGTGAGCTATGAGAAAGCGCCACGCTTCCCGAAG
GGAGAAAGGCGGACAGGTATCCGGTAAGCGGCAGGGTCGGAACAGGAGAGCGCACGAGGGAGCT
TCCAGGGGGAAACGCCTGGTATCTTTATAGTCCTGTCGGGTTTCGCCACCTCTGACTTGAGCGTCGA
TTTTTGTGATGCTCGTCAGGGGGGCGGAGCCTATGGAAAAACGCCAGCAACGCGGCCTTTTTACGG
TTCCTGGCCTTTTGCTGGCCTTTTGCTCACATGT

pTRE3G-GFP with Neo^R

CTCGAGTTTACTCCCTATCAGTGATAGAGAACGTATGAAGAGTTTACTCCCTATCAGTGATAGAGA
ACGTATGCAGACTTTACTCCCTATCAGTGATAGAGAACGTATAAGGAGTTTACTCCCTATCAGTGA
TAGAGAACGTATGACCAGTTTACTCCCTATCAGTGATAGAGAACGTATCTACAGTTTACTCCCTATC
AGTGATAGAGAACGTATATCCAGTTTACTCCCTATCAGTGATAGAGAACGTATAAGCTTTAGGCGT
GTACGGTGGGCGCCTATAAAAGCAGAGCTCGTTTAGTGAACCGTCAGATCGCCTGGAGCAATTCCA
CAACACTTTTGTCTTATACCAACTTTCCGTACCACTTCCTACCCTCGTAAAGTCGACATGGTGAGCA

AGGGCGAGGAGCTGTTCACCGGGGTGGTGCCCATCCTGGTCGAGCTGGACGGCGACGTAAACGGC
CACAAGTTCAGCGTGTCCGGCGAGGGCGAGGGCGATGCCACCTACGGCAAGCTGACCCTGAAGTT
CATCTGCACCACCGGCAAGCTGCCCGTGCCCTGGCCCACCCTCGTGACCACCCTGACCTACGGCGT
GCAGTGCTTCAGCCGCTACCCCGACCACATGAAGCAGCACGACTTCTTCAAGTCCGCCATGCCCGA
AGGCTACGTCCAGGAGCGCACCATCTTCTTCAAGGACGACGGCAACTACAAGACCCGCGCCGAGG
TGAAGTTCGAGGGCGACACCCTGGTGAACCGCATCGAGCTGAAGGGCATCGACTTCAAGGAGGAC
GGCAACATCCTGGGGCACAAGCTGGAGTACAACTACAACAGCCACAACGTCTATATCATGGCCGA
CAAGCAGAAGAACGGCATCAAGGTGAACTTCAAGATCCGCCACAACATCGAGGACGGCAGCGTGC
AGCTCGCCGACCACTACCAGCAGAACACCCCCATCGGCGACGGCCCCGTGCTGCTGCCCGACAACC
ACTACCTGAGCACCCAGTCCGCCCTGAGCAAAGACCCCAACGAGAAGCGCGATCACATGGTCCTG
CTGGAGTTCGTGACCGCCGCCGGGATCACTCTCGGCATGGACGAGCTGTACAAGTAAAGATCTATC
GATCGGCCGGCCCCTCTCCCTCCCCCCCCCCCTAACGTTACTGGCCGAAGCCGCTTGGAATAAGGC
CGGTGTGCGTTTGTCTATATGTTATTTTCCACCATATTGCCGTCTTTTGGCAATGTGAGGGCCCGGA
AACCTGGCCCTGTCTTCTTGACGAGCATTCCTAGGGGTCTTTCCCCTCTCGCCAAAGGAATGCAAGG
TCTGTTGAATGTCGTGAAGGAAGCAGTTCCTCTGGAAGCTTCTTGAAGACAAACAACGTCTGTAGC
GACCCTTTGCAGGCAGCGGAACCCCCCACCTGGCGACAGGTGCCTCTGCGGCCAAAAGCCACGTGT
ATAAGATACACCTGCAAAGGCGGCACAACCCCAGTGCCACGTTGTGAGTTGGATAGTTGTGGAAA
GAGTCAAATGGCTCTCCTCAAGCGTATTCAACAAGGGGCTGAAGGATGCCCAGAAGGTACCCCATT
GTATGGGATCTGATCTGGGGCCTCGGTACACATGCTTTACATGTGTTTAGTCGAGGTTAAAAAAAC
GTCTAGGCCCCCCGAACCACGGGGACGTGGTTTTCCTTTGAAAAACACGATGATAATATGGCCACA
ACCGGGCCGGATATCACGCGTCATATGGCTAGCCTGCAGGGATCCAATGTAACTGTATTCAGCGAT
GACGAAATTCTTAGCTATTGTAATACTCTAGAGGATCTTTGTGAAGGAACCTTACTTCTGTGGTGTG
ACATAATTGGACAAACTACCTACAGAGATTTAAAGCTCTAAGGTAAATATAAAATTTTTAAGTGTA
TAATGTGTTAAACTACTGATTCTAATTGTTTGTGTATTTTAGATTCCAACCTATGGAACTGATGAAT
GGGAGCAGTGGTGGAATGCCTTTAATGAGGAAAACCTGTTTTGCTCAGAAGAAATGCCATCTAGTG
ATGATGAGGCTACTGCTGACTCTCAACATTCTACTCCTCCAAAAAAGAAGAGAAAGGTAGAAGAC
CCCAAGGACTTTCCTTCAGAATTGCTAAGTTTTTTGAGTCATGCTGTGTTTAGTAATAGAACTCTTG
CTTGCTTTGCTATTTACACCACAAAGGAAAAAGCTGCACTGCTATACAAGAAAATTATGGAAAAAT
ATTCTGTAACCTTTATAAGTAGGCATAACAGTTATAATCATAACATACTGTTTTTTCTTACTCCACA
CAGGCATAGAGTGTCTGCTATTAATAACTATGCTCAAAAATTGTGTACCTTTAGCTTTTTAATTTGT
AAAGGGGTTAATAAGGAATATTTGATGTATAGTGCCTTGACTAGAGATCATAATCAGCCATACCAC
ATTTGTAGAGGTTTTACTTGCTTTAAAAAACCTCCCACACCTCCCCCTGAACCTGAAACATAAAATG
AATGCAATTGTTGTTGTTAACTTGTTTATTGCAGCTTATAATGGTTACAAATAAAGCAATAGCATCA
CAAATTTCACAAATAAAGCATTTTTTTCACTGCATTCTAGTTGTGGTTTGTCCAAACTCATCAATGT
ATCTTATCATGTCTGCGGCTCTAGAGCTGCATTAATGAATCGGCCAACGCGCGGGGAGAGGCGGTT
TGCGTATTGGGCGCTCTTCCGCTTCCTCGCTCACTGACTCGCTGCGCTCGGTCGTTCGGCTGCGGCG
AGCGGTATCAGCTCACTCAAAGGCGGTAATACGGTTATCCACAGAATCAGGGGATAACGCAGGAA
AGAACATGTGAGCAAAAGGCCAGCAAAAGGCCAGGAACCGTAAAAAGGCCGCGTTGCTGGCGTTT
TTCCATAGGCTCCGCCCCCCTGACGAGCATCACAAAAATCGACGCTCAAGTCAGAGGTGGCGAAAC
CCGACAGGACTATAAAGATACCAGGCGTTTCCCCCTGGAAGCTCCCTCGTGCGCTCTCCTGTTCCG
ACCCTGCCGCTTACCGGATACCTGTCCGCCTTTCTCCCTTCGGGAAGCGTGGCGCTTTCTCATAGCT
CACGCTGTAGGTATCTCAGTTCGGTGTAGGTCGTTCGCTCCAAGCTGGGCTGTGTGCACGAACCCC
CCGTTCAGCCCGACCGCTGCGCCTTATCCGGTAACTATCGTCTTGAGTCCAACCCGGTAAGACACG
ACTTATCGCCACTGGCAGCAGCCACTGGTAACAGGATTAGCAGAGCGAGGTATGTAGGCGGTGCT
ACAGAGTTCTTGAAGTGGTGGCCTAACTACGGCTACACTAGAAGAACAGTATTTGGTATCTGCGCT
CTGCTGAAGCCAGTTACCTTCGGAAAAAGAGTTGGTAGCTCTTGATCCGGCAAACAAACCACCGCT
GGTAGCGGTGGTTTTTTTGTTTGCAAGCAGCAGATTACGCGCAGAAAAAAAGGATCTCAAGAAGAT
CCTTTGATCTTTTCTACGGGGTCTGACGCTCAGTGGAACGAAAACTCACGTTAAGGGATTTTGGTCA
TGAGATTATCAAAAAGGATCTTCACCTAGATCCTTTTAAATTAAAAATGAAGTTTTAAATCAATCTA
AAGTATATATGAGTAACCTGAGGCTATGGCAGGGCCTGCCGCCCCGACGTTGGCTGCGAGCCCTGG
GCCTTCACCCGAACTTGGGGGGTGGGGTGGGGAAAAGGAAGAAACGCGGGCGTATTGGCCCCAAT
GGGGTCTCGGTGGGGTATCGACAGAGTGCCAGCCCTGGGACCGAACCCCGCGTTTATGAACAAAC
GACCCAACACCGTGCGTTTTATTCTGTCTTTTTATTGCCGTCATAGCGCGGGTTCCTTCCGGTATTGT
CTCCTTCCGTGTTTCAGTTAGCCTCCCCCTAGGGTGGGCGAAGAACTCCAGCATGAGATCCCCGCG
CTGGAGGATCATCCAGCCGGCGTCCCGGAAAACGATTCCGAAGCCCAACCTTTCATAGAAGGCGG
CGGTGGAATCGAAATCTCGTGATGGCAGGTTGGGCGTCGCTTGGTCGGTCATTTCGAACCCCAGAG

TCCCGCTCAGAAGAACTCGTCAAGAAGGCGATAGAAGGCGATGCGCTGCGAATCGGGAGCGGCGA
TACCGTAAAGCACGAGGAAGCGGTCAGCCCATTCGCCGCCAAGCTCTTCAGCAATATCACGGGTAG
CCAACGCTATGTCCTGATAGCGGTCCGCCACACCCAGCCGGCCACAGTCGATGAATCCAGAAAAGC
GGCCATTTTCCACCATGATATTCGGCAAGCAGGCATCGCCATGGGTCACGACGAGATCCTCGCCGT
CGGGCATGCTCGCCTTGAGCCTGGCGAACAGTTCGGCTGGCGCGAGCCCCTGATGCTCTTCGTCCA
GATCATCCTGATCGACAAGACCGGCTTCCATCCGAGTACGTGCTCGCTCGATGCGATGTTTCGCTTG
GTGGTCGAATGGGCAGGTAGCCGGATCAAGCGTATGCAGCCGCCGCATTGCATCAGCCATGATGG
ATACTTTCTCGGCAGGAGCAAGGTGAGATGACAGGAGATCCTGCCCCGGCACTTCGCCCAATAGCA
GCCAGTCCCTTCCCGCTTCAGTGACAACGTCGAGCACAGCTGCGCAAGGAACGCCCGTCGTGGCCA
GCCACGATAGCCGCGCTGCCTCGTCTTGCAGTTCATTCAGGGCACCGGACAGGTCGGTCTTGACAA
AAAGAACCGGGCGCCCCTGCGCTGACAGCCGGAACACGGCGGCATCAGAGCAGCCGATTGTCTGT
TGTGCCCAGTCATAGCCGAATAGCCTCTCCACCCAAGCGGCCGGAGAACCTGCGTGCAATCCATCT
TGTTCAATCATGCGAAACGATCCTCATCCTGTCTCTTGATCGATCTTTGCAAAAGCCTAGGCCTCCA
AAAAAGCCTCCTCACTACTTCTGGAATAGCTCAGAGGCCGAGGCGGCCTCGGCCTCTGCATAAATA
AAAAAAATTAGTCAGCCATGGGGCGGAGAATGGGCGGAACTGGGCGGAGTTAGGGGCGGGATGG
GCGGAGTTAGGGGCGGGACTATGGTTGCTGACTAATTGAGATGCATGCTTTGCATACTTCTGCCTG
CTGGGGAGCCTGGGGACTTTCCACACCTGGTTGCTGACTAATTGAGATGCATGCTTTGCATACTTCT
GCCTGCTGGGGAGCCTGGGGACTTTCCACACCCTAACTGACACACATTCCACAGCTGGTTCTTTCCG
CCTCAGGACTCTTCCTTTTTCAATATTATTGAAGCATTTATCAGGGTTATTGTCTCATGAGCGGATA
CATATTTGAATGTATTTAGAAAAATAAACAAATAGGGGTTCCGCGCACATTTCCCCGAAAAGTGCC
ACCTGACGTCTAAGAAACCATTATTATCATGACATTAACCTATAAAAATAGGCGTATCACGAGGCC
CTTTCGTCTTCAAGAATTC

pTRE3G-GFP-uL10 with Neo^R

CTCGAGTTTACTCCCTATCAGTGATAGAGAACGTATGAAGAGTTTACTCCCTATCAGTGATAGAGA
ACGTATGCAGACTTTACTCCCTATCAGTGATAGAGAACGTATAAGGAGTTTACTCCCTATCAGTGA
TAGAGAACGTATGACCAGTTTACTCCCTATCAGTGATAGAGAACGTATCTACAGTTTACTCCCTATC
AGTGATAGAGAACGTATATCCAGTTTACTCCCTATCAGTGATAGAGAACGTATAAGCTTTAGGCGT
GTACGGTGGGCGCCTATAAAAGCAGAGCTCGTTTAGTGAACCGTCAGATCGCCTGGAGCAATTCCA
CAACACTTTTGTCTTATACCAACTTTCCGTACCACTTCCTACCCTCGTAAAGTCGACATGGTGAGCA
AGGGCGAGGAGCTGTTCACCGGGGTGGTGCCCATCCTGGTCGAGCTGGACGGCGACGTAAACGGC
CACAAGTTCAGCGTGTCCGGCGAGGGCGAGGGCGATGCCACCTACGGCAAGCTGACCCTGAAGTT
CATCTGCACCACCGGCAAGCTGCCCGTGCCCTGGCCCACCCTCGTGACCACCCTGACCTACGGCGT
GCAGTGCTTCAGCCGCTACCCCGACCACATGAAGCAGCACGACTTCTTCAAGTCCGCCATGCCCGA
AGGCTACGTCCAGGAGCGCACCATCTTCTTCAAGGACGACGGCAACTACAAGACCCGCGCCGAGG
TGAAGTTCGAGGGCGACACCCTGGTGAACCGCATCGAGCTGAAGGGCATCGACTTCAAGGAGGAC
GGCAACATCCTGGGGCACAAGCTGGAGTACAACTACAACAGCCACAACGTCTATATCATGGCCGA
CAAGCAGAAGAACGGCATCAAGGTGAACTTCAAGATCCGCCACAACATCGAGGACGGCAGCGTGC
AGCTCGCCGACCACTACCAGCAGAACACCCCCATCGGCGACGGCCCCGTGCTGCTGCCCGACAACC
ACTACCTGAGCACCCAGTCCGCCCTGAGCAAAGACCCCAACGAGAAGCGCGATCACATGGTCCTG
CTGGAGTTCGTGACCGCCGCCGGGATCACTCTCGGCATGGACGAGCTGTACAAGTCCGGACTCAGA
TCTCGAGCTCAAGCTTCGAATTCTATGCCCAGGGAAGACAGGGCGACCTGGAAGTCCAACTACTTC
CTTAAGATCATCCAACTATTGGATGATTATCCGAAATGTTTCATTGTGGGAGCAGACAATGTGGGC
TCCAAGCAGATGCAGCAGATCCGCATGTCCCTTCGCGGGAAGGCTGTGGTGCTGATGGGCAAGAA
CACCATGATGCGCAAGGCCATCCGAGGGCACCTGGAAAACAACCCAGCTCTGGAGAAACTGCTGC
CTCATATCCGGGGGAATGTGGGCTTTGTGTTCACCAAGGAGGACCTCACTGAGATCAGGGACATGT
TGCTGGCCAATAAGGTGCCAGCTGCTGCCCGTGCTGGTGCCATTGCCCCATGTGAAGTCACTGTGC
CAGCCCAGAACACTGGTCTCGGGCCCGAGAAGACCTCCTTTTTCCAGGCTTTAGGTATCACCACTA
AAATCTCCAGGGGCACCATTGAAATCCTGAGTGATGTGCAGCTGATCAAGACTGGAGACAAAGTG
GGAGCCAGCGAAGCCACGCTGCTGAACATGCTCAACATCTCCCCCTTCTCCTTTGGGCTGGTCATCC
AGCAGGTGTTCGACAATGGCAGCATCTACAACCCTGAAGTGCTTGATATCACAGAGGAAACTCTGC
ATTCTCGCTTCCTGGAGGGTGTCCGCAATGTTGCCAGTGTCTGTCTGCAGATTGGCTACCCAACTGT
TGCATCAGTACCCCATTCTATCATCAACGGGTACAAACGAGTCCTGGCCTTGTCTGTGGAGACGGA
TTACACCTTCCCACTTGCTGAAAAGGTCAAGGCCTTCTTGGCTGATCCATCTGCCTTTGTGGCTGCT

GCCCCTGTGGCTGCTGCCACCACAGCTGCTCCTGCTGCTGCTGCAGCCCCAGCTAAGGTTGAAGCC
AAGGAAGAGTCGGAGGAGTCGGACGAGGATATGGGATTTGGTCTCTTTGACTAAAGATCTATCGAT
CGGCCGGCCCCTCTCCCTCCCCCCCCCCTAACGTTACTGGCCGAAGCCGCTTGGAATAAGGCCGG
TGTGCGTTTGTCTATATGTTATTTTCCACCATATTGCCGTCTTTTGGCAATGTGAGGGCCCGGAAAC
CTGGCCCTGTCTTCTTGACGAGCATTCCTAGGGGTCTTTCCCCTCTCGCCAAAGGAATGCAAGGTCT
GTTGAATGTCGTGAAGGAAGCAGTTCCTCTGGAAGCTTCTTGAAGACAAACAACGTCTGTAGCGAC
CCTTTGCAGGCAGCGGAACCCCCCACCTGGCGACAGGTGCCTCTGCGGCCAAAAGCCACGTGTATA
AGATACACCTGCAAAGGCGGCACAACCCCAGTGCCACGTTGTGAGTTGGATAGTTGTGGAAAGAG
TCAAATGGCTCTCCTCAAGCGTATTCAACAAGGGGCTGAAGGATGCCCAGAAGGTACCCCATTGTA
TGGGATCTGATCTGGGGCCTCGGTACACATGCTTTACATGTGTTTAGTCGAGGTTAAAAAAACGTC
TAGGCCCCCCGAACCACGGGGACGTGGTTTTCCTTTGAAAAACACGATGATAATATGGCCACAACC
GGGCCGGATATCACGCGTCATATGGCTAGCCTGCAGGGATCCAATGTAACTGTATTCAGCGATGAC
GAAATTCTTAGCTATTGTAATACTCTAGAGGATCTTTGTGAAGGAACCTTACTTCTGTGGTGTGACA
TAATTGGACAAACTACCTACAGAGATTTAAAGCTCTAAGGTAAATATAAAATTTTTAAGTGTATAA
TGTGTTAAACTACTGATTCTAATTGTTTGTGTATTTTAGATTCCAACCTATGGAACTGATGAATGGG
AGCAGTGGTGGAATGCCTTTAATGAGGAAAACCTGTTTTGCTCAGAAGAAATGCCATCTAGTGATG
ATGAGGCTACTGCTGACTCTCAACATTCTACTCCTCCAAAAAAGAAGAGAAAGGTAGAAGACCCC
AAGGACTTTCCTTCAGAATTGCTAAGTTTTTTGAGTCATGCTGTGTTTAGTAATAGAACTCTTGCTT
GCTTTGCTATTTACACCACAAAGGAAAAAGCTGCACTGCTATACAAGAAAATTATGGAAAAATATT
CTGTAACCTTTATAAGTAGGCATAACAGTTATAATCATAACATACTGTTTTTTCTTACTCCACACAG
GCATAGAGTGTCTGCTATTAATAACTATGCTCAAAAATTGTGTACCTTTAGCTTTTTAATTTGTAAA
GGGGTTAATAAGGAATATTTGATGTATAGTGCCTTGACTAGAGATCATAATCAGCCATACCACATT
TGTAGAGGTTTTACTTGCTTTAAAAAACCTCCCACACCTCCCCCTGAACCTGAAACATAAAATGAA
TGCAATTGTTGTTGTTAACTTGTTTATTGCAGCTTATAATGGTTACAAATAAAGCAATAGCATCACA
AATTTCACAAATAAAGCATTTTTTTCACTGCATTCTAGTTGTGGTTTGTCCAAACTCATCAATGTAT
CTTATCATGTCTGCGGCTCTAGAGCTGCATTAATGAATCGGCCAACGCGCGGGGAGAGGCGGTTTG
CGTATTGGGCGCTCTTCCGCTTCCTCGCTCACTGACTCGCTGCGCTCGGTCGTTCGGCTGCGGCGAG
CGGTATCAGCTCACTCAAAGGCGGTAATACGGTTATCCACAGAATCAGGGGATAACGCAGGAAAG
AACATGTGAGCAAAAGGCCAGCAAAAGGCCAGGAACCGTAAAAAGGCCGCGTTGCTGGCGTTTTT
CCATAGGCTCCGCCCCCCTGACGAGCATCACAAAAATCGACGCTCAAGTCAGAGGTGGCGAAACC
CGACAGGACTATAAAGATACCAGGCGTTTCCCCCTGGAAGCTCCCTCGTGCGCTCTCCTGTTCCGA
CCCTGCCGCTTACCGGATACCTGTCCGCCTTTCTCCCTTCGGGAAGCGTGGCGCTTTCTCATAGCTC
ACGCTGTAGGTATCTCAGTTCGGTGTAGGTCGTTCGCTCCAAGCTGGGCTGTGTGCACGAACCCCC
CGTTCAGCCCGACCGCTGCGCCTTATCCGGTAACTATCGTCTTGAGTCCAACCCGGTAAGACACGA
CTTATCGCCACTGGCAGCAGCCACTGGTAACAGGATTAGCAGAGCGAGGTATGTAGGCGGTGCTAC
AGAGTTCTTGAAGTGGTGGCCTAACTACGGCTACACTAGAAGAACAGTATTTGGTATCTGCGCTCT
GCTGAAGCCAGTTACCTTCGGAAAAAGAGTTGGTAGCTCTTGATCCGGCAAACAAACCACCGCTGG
TAGCGGTGGTTTTTTTGTTTGCAAGCAGCAGATTACGCGCAGAAAAAAAGGATCTCAAGAAGATCC
TTTGATCTTTTCTACGGGGTCTGACGCTCAGTGGAACGAAAACTCACGTTAAGGGATTTTGGTCATG
AGATTATCAAAAAGGATCTTCACCTAGATCCTTTTAAATTAAAAATGAAGTTTTAAATCAATCTAA
AGTATATATGAGTAACCTGAGGCTATGGCAGGGCCTGCCGCCCCGACGTTGGCTGCGAGCCCTGGG
CCTTCACCCGAACTTGGGGGGTGGGGTGGGGAAAAGGAAGAAACGCGGGCGTATTGGCCCCAATG
GGGTCTCGGTGGGGTATCGACAGAGTGCCAGCCCTGGGACCGAACCCCGCGTTTATGAACAAACG
ACCCAACACCGTGCGTTTTATTCTGTCTTTTTATTGCCGTCATAGCGCGGGTTCCTTCCGGTATTGTC
TCCTTCCGTGTTTCAGTTAGCCTCCCCCTAGGGTGGGCGAAGAACTCCAGCATGAGATCCCCGCGCT
GGAGGATCATCCAGCCGGCGTCCCGGAAAACGATTCCGAAGCCCAACCTTTCATAGAAGGCGGCG
GTGGAATCGAAATCTCGTGATGGCAGGTTGGGCGTCGCTTGGTCGGTCATTTCGAACCCCAGAGTC
CCGCTCAGAAGAACTCGTCAAGAAGGCGATAGAAGGCGATGCGCTGCGAATCGGGAGCGGCGATA
CCGTAAAGCACGAGGAAGCGGTCAGCCCATTCGCCGCCAAGCTCTTCAGCAATATCACGGGTAGCC
AACGCTATGTCCTGATAGCGGTCCGCCACACCCAGCCGGCCACAGTCGATGAATCCAGAAAAGCG
GCCATTTTCCACCATGATATTCGGCAAGCAGGCATCGCCATGGGTCACGACGAGATCCTCGCCGTC
GGGCATGCTCGCCTTGAGCCTGGCGAACAGTTCGGCTGGCGCGAGCCCCTGATGCTCTTCGTCCAG
ATCATCCTGATCGACAAGACCGGCTTCCATCCGAGTACGTGCTCGCTCGATGCGATGTTTCGCTTGG
TGGTCGAATGGGCAGGTAGCCGGATCAAGCGTATGCAGCCGCCGCATTGCATCAGCCATGATGGAT
ACTTTCTCGGCAGGAGCAAGGTGAGATGACAGGAGATCCTGCCCCGGCACTTCGCCCAATAGCAGC
CAGTCCCTTCCCGCTTCAGTGACAACGTCGAGCACAGCTGCGCAAGGAACGCCCGTCGTGGCCAGC

CACGATAGCCGCGCTGCCTCGTCTTGCAGTTCATTCAGGGCACCGGACAGGTCGGTCTTGACAAAA
AGAACCGGGCGCCCCTGCGCTGACAGCCGGAACACGGCGGCATCAGAGCAGCCGATTGTCTGTTG
TGCCCAGTCATAGCCGAATAGCCTCTCCACCCAAGCGGCCGGAGAACCTGCGTGCAATCCATCTTG
TTCAATCATGCGAAACGATCCTCATCCTGTCTCTTGATCGATCTTTGCAAAAGCCTAGGCCTCCAAA
AAAGCCTCCTCACTACTTCTGGAATAGCTCAGAGGCCGAGGCGGCCTCGGCCTCTGCATAAATAAA
AAAAATTAGTCAGCCATGGGGCGGAGAATGGGCGGAACTGGGCGGAGTTAGGGGCGGGATGGGC
GGAGTTAGGGGCGGGACTATGGTTGCTGACTAATTGAGATGCATGCTTTGCATACTTCTGCCTGCT
GGGGAGCCTGGGGACTTTCCACACCTGGTTGCTGACTAATTGAGATGCATGCTTTGCATACTTCTGC
CTGCTGGGGAGCCTGGGGACTTTCCACACCCTAACTGACACACATTCCACAGCTGGTTCTTTCCGCC
TCAGGACTCTTCCTTTTTCAATATTATTGAAGCATTTATCAGGGTTATTGTCTCATGAGCGGATACA
TATTTGAATGTATTTAGAAAAATAAACAAATAGGGGTTCCGCGCACATTTCCCCGAAAAGTGCCAC
CTGACGTCTAAGAAACCATTATTATCATGACATTAACCTATAAAAATAGGCGTATCACGAGGCCCT
TTCGTCTTCAAGAATTC

Tables:

Table S1. Primers used for cloning and sequencing

**PRIMERS USED FOR CLONING**		
**GIBSON (TET3G T2A PURO FUSION)**	**VECTOR Fw**	ACCTTGACATGCTCCCCGGGGAATTCGGCAGTGGAGAGG
	**VECTOR Rev**	GCTCTTGTCCAGTCTAGACATGGTGGCACCGGTCCAACC
	**INSERT Fw**	TCTAGACTGGACAAGAGCAAAGTC
	**INSERT Rev**	CCCGGGGAGCATGTCAAGGTCA
**REPLACR (KAN/NEO CASSETTE)**	**NEO/Fw**	CGCTCATGAGACAATAACCCTG
	**NEO/Rev**	GGTCTGACGCTCAGTGGAACG
**GIBSON (TRE3G GFP-uL10 KAN/NEO)**	**VECTOR Fw**	GATTTGGTCTCTTTGACTAAAGATCTATCGATCGGCCGGATATC
	**VECTOR Rev**	CCTCGCCCTTGCTCACCATGTCGACTTTACGAGGGTAGGAAGTG
	**INSERT Fw**	ATGGTGAGCAAGGGCGAGGAGC
	**INSERT Rev**	TTAGTCAAAGAGACCAAATCCC
**TRE3G GFP KAN/NEO**	**Fw**	CGCGTCGACATGGTGAGCAAGGGCGAGGAG (SalI)
	**Rev**	GCGCATATGTTACTTGTACAGCTCGTCCAT (NdeI)
**PRIMERS USED FOR SEQUENCING**		
**TET3G T2A PURO FUSION**	**Checking For (hybrid intron)**	GGCTGTAATTAGCTGAGCAAGAGG
**KAN/NEO CASSETTE**	**Neo-F**	CGTTGGCTACCCGTGATATT
	**Neo-R**	GCCCAGTCATAGCCGAATAG
**TRE3G GFP-uL10 KAN/NEO**	**EGFPC seq primer**	CATGGTCCTGCTGGAGTTCGTG
	**TRE F**	GCAGAGCTCGTTTAGTGAACC
**TRE3G GFP KAN/NEO**	**GFPN seq primer**	CTTGTACAGCTCGTCCATGCC
